# Partial Prion Cross-Seeding between Fungal and Mammalian Amyloid Signaling Motifs

**DOI:** 10.1128/mBio.02782-20

**Published:** 2021-02-09

**Authors:** Thierry Bardin, Asen Daskalov, Sophie Barrouilhet, Alexandra Granger-Farbos, Bénédicte Salin, Corinne Blancard, Brice Kauffmann, Sven J. Saupe, Virginie Coustou

**Affiliations:** a Non-Self Recognition in Fungi, Institut de Biochimie et de Génétique Cellulaire (CNRS UMR 5095), Université de Bordeaux, Bordeaux, France; b IECB, UMS 3033, US 001, CNRS, Université de Bordeaux, Pessac, France; National Institutes of Health

**Keywords:** RIP1, RIP3, RHIM, amyloid, necroptosis, prion, programmed cell death

## Abstract

In filamentous fungi, NLR-based signalosomes activate downstream membrane-targeting cell death-inducing proteins by a mechanism of amyloid templating. In the species Podospora anserina, two such signalosomes, NWD2/HET-S and FNT1/HELLF, have been described. An analogous system involving a distinct amyloid signaling motif, termed PP, was also identified in the genome of the species Chaetomium globosum and studied using heterologous expression in Podospora anserina. The PP motif bears resemblance to the RIP homotypic interaction motif (RHIM) and to RHIM-like motifs controlling necroptosis in mammals and innate immunity in flies. We identify here a third NLR signalosome in Podospora anserina comprising a PP motif and organized as a two-gene cluster encoding an NLR and an HELL domain cell death execution protein termed HELLP. We show that the PP motif region of HELLP forms a prion we term [π] and that [π] prions trigger the cell death-inducing activity of full-length HELLP. We detect no prion cross-seeding between HET-S, HELLF, and HELLP amyloid motifs. In addition, we find that, like PP motifs, RHIMs from human RIP1 and RIP3 kinases are able to form prions in Podospora and that [π] and [Rhim] prions partially cross-seed. Our study shows that Podospora anserina displays three independent cell death-inducing amyloid signalosomes. Based on the described functional similarity between RHIM and PP, it appears likely that these amyloid motifs constitute evolutionarily related cell death signaling modules.

## Abstract (precis)

Amyloids are β-sheet-rich protein polymers that can be pathological or display a variety of biological roles. In filamentous fungi, specific immune receptors activate programmed cell death execution proteins through a process of amyloid templating akin to prion propagation.

## INTRODUCTION

**FIG 1:**
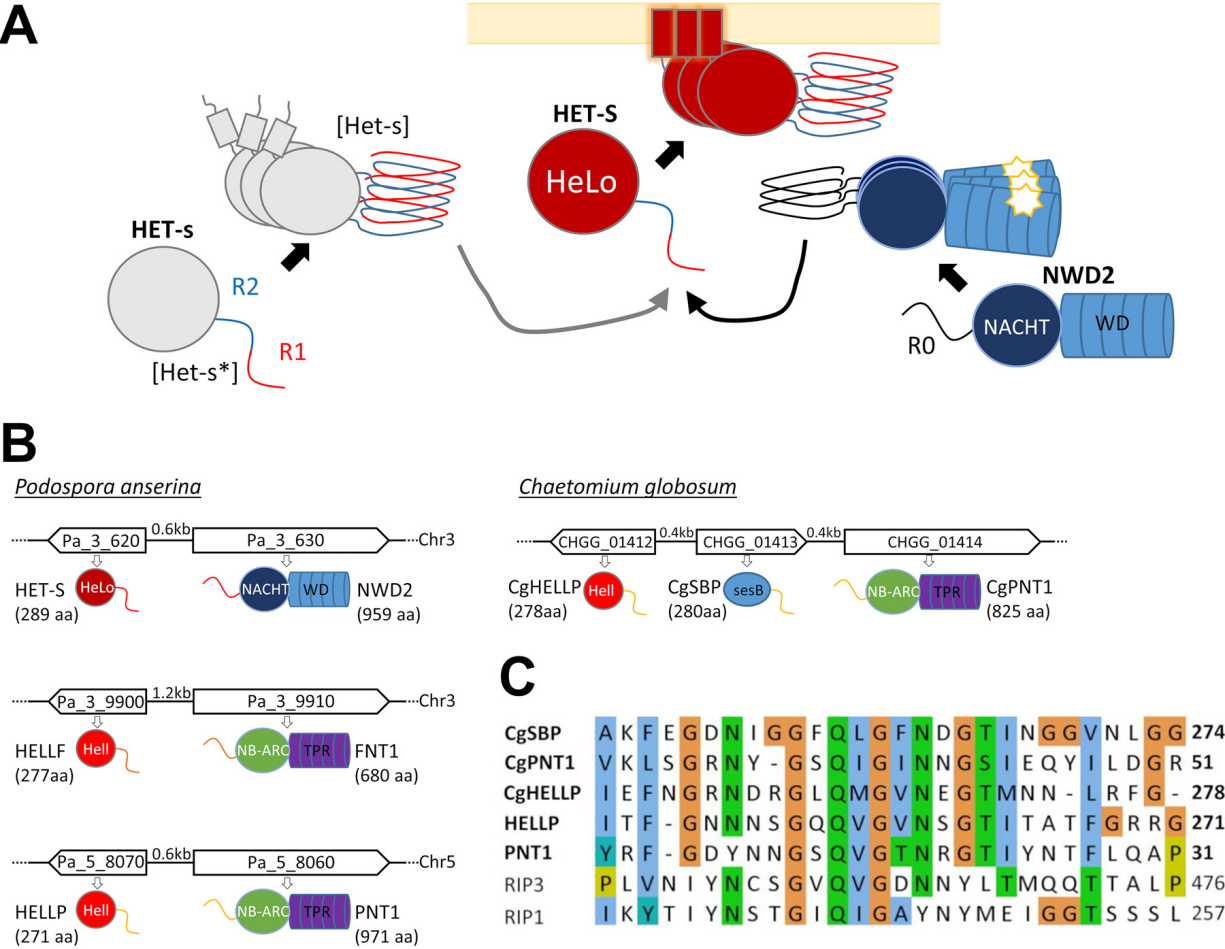
NRL signalosomes in fungi. (A) Schematic representation of the NWD2/HET-S signalosome and the [Het-s]/HET-S incompatibility system. HET-S is a cell death execution protein with the HeLo pore-forming domain and a C-terminal prion-forming domain comprising two pseudorepeats (R1 and R2) that is natively unfolded in the soluble state of the protein. HET-S can be activated by two distinct pathways. In [Het-s]/HET-S incompatibility, HET-s, an allelic variant of HET-S with an inactive HeLo domain, can switch from a soluble state ([Het-s]) to a prion state ([Het-s]). The prion-forming domain of HET-s converts the corresponding domain of HET-S to the amyloid fold, inducing activation of the HeLo domain, which inserts into the membrane by formation of a N-terminal transmembrane helix and causes cell death. NWD2 is an NLR with a NACHT domain and WD repeats and an N-terminal region homologous to the elementary PFD repeats (R0). Upon binding of a ligand to the WD repeats, the NLR is proposed to oligomerize. Oligomerization leads to amyloid formation in the R0 region, which is able to convert the corresponding region of HET-S and induce its toxicity. (B) Genome organization and domain architecture of the *het-s*–*nwd2*, *hellf*-*fnt1*, and *hellp*-*pnt1* gene clusters of *P. anserina* and the *hellp-sbp-pnt1* cluster of *C. globosum*. Chromosome, gene orientation, and intergenic distances and protein size are given. These loci are composed of two adjacent genes encoding an effector protein and an NLR receptor. The effector proteins are composed of a PFD and an HeLo or HELL domain. The NLRs are composed of a PFD, an NB-ARC, or NACHT nucleotide binding and oligomerization domain and of WD repeats or TPRs (repeat numbers are variable in different wild-type isolates and varied from 2 to 11, 4 to 11, and 4 to 10 in *nwd2*, *fnt1*, and *pnt1*, respectively). (C) Alignment of the PP motifs of HELLP, PNT1, CgHELLP, CgSBP, and CgPNT1, along with the RHIMs of human RIP1 and RIP3.

Prions are protein polymers that behave as infectious entities by propagating their amyloid structural state (1, 2). In addition to disease-causing prions in mammals, prions have been identified in fungi as infectious, cytoplasmic, non-Mendelian genetic elements (3). One such prion is [Het-s] of the filamentous fungus Podospora anserina. [Het-s] functions in a nonself recognition process known as heterokaryon incompatibility. The *het-s* gene exists as two incompatible allelic variants termed *het-s* and *het-S* (Fig. 1A). The HET-s protein, when assembled into the self-propagating prion form [Het-s], confers incompatibility to the HET-S protein variant. Coexpression of the [Het-s] prion with HET-S leads to cell death. At the macroscopic level, the incompatibility reaction leads to the formation of an abnormal contact line between the strains termed “barrage”. The soluble nonprion form confers a phenotype termed [Het-s*] that is compatible with HET-S (4, 5). [Het-s*] strains acquire the prion state either spontaneously at a low frequency or systematically after fusion with a prion-infected strain. HET-s and HET-S are highly homologous variants of the same protein displaying two domains, a C-terminal prion-forming domain (PFD) necessary and sufficient for prion propagation and an N-terminal α-helical globular domain named HeLo responsible for cell death-inducing activity (6, 7). The HET-s PFD is natively unfolded in the soluble state and, upon prion formation, assembles into a specific cross-β amyloid structure (6, 8–10). The HET-s β-solenoid fold is composed of 2 rungs of β-strands per monomer, comprising two 21-amino-acid-long imperfect repeats (R1 and R2) connected by a flexible loop (9, 11). In [Het-s]/HET-S incompatibility, cell death is triggered when the [Het-s] PFD templates conversion of the HET-S PFD region into the β-solenoid fold, which in turn induces refolding of the HET-S HeLo domain that acquires pore-forming activity by exposing an N-terminal transmembrane helix targeting the cell membrane (7, 12). In this incompatibility system, HET-S acts as a cell death execution protein, and [Het-s] triggers its activation. While displaying a functional PFD, HET-S, in contrast to HET-s, cannot form a prion propagating *in vivo* because activation of its HeLo domain leads to toxicity. In HET-s, in turn, cell death-inducing activity of the HeLo domain is compromised by a point mutation in the transmembrane helix region. In other words, HET-s can form a prion because its HeLo domain is inactivated (6, 7, 13).

Further work on the [Het-s] system revealed that this incompatibility system is evolutionary derived from a regulated cell death (RCD) pathway in which HET-S is activated by a specific NLR (Nod-like receptor) (14). NLR receptors are intracellular receptors that control immune defense and RCD pathways in animals, plants, and fungi and function by ligand-induced oligomerization (15, 16). The NWD2 NLR receptor is encoded by the gene immediately adjacent to *het-S* in the P. anserina genome and displays a central NACHT nucleotide binding and oligomerization domain and a C-terminal WD repeat domain. The N-terminal region of NWD2 displays a short region of homology to the R1 and R2 motifs of HET-s. This region, designated R0, is able to adopt an *het-s*-like fold. Ligand-induced oligomerization of NWD2 allows for spatial clustering of R0 motifs and cooperative nucleation of the β-solenoid fold, which then templates conversion of the homologous domain in HET-S and activation of the HeLo domain (14, 17, 18). More generally, it is proposed that a fraction of the NLR receptors in fungi activate their cognate effector domains by a mechanism of amyloid templating by which the amyloid fold formed after oligomerization of the NLR receptor is transmitted through heterotypic interactions to the amyloid motif found in the C-terminal region of the effector protein (19). The *nwd2*-*het-S* gene pair was used as a paradigm in bioinformatic screens to identify additional NLR/effector pairs functioning through amyloid templating in fungi (17, 20). These approaches resulted in particular in the identification of five *het-s*-related amyloid motif families (HRAMs) and in the characterization in Podospora anserina of a distant homolog of HET-S termed HELLF and belonging the HRAM5 family (21). HELLF encodes a protein with a HeLo-like N-terminal domain and a C-terminal PFD with two repeated submotifs that share only 17% identity with the HET-s PFD. The HeLo-like domain shows distant homology to the HET-S HeLo domain but also to the 4HB pore-forming domain of the MLKL protein (22), the terminal effector protein of necroptotic death in mammals, which led to the proposition that this form of fungal RCD is related to mammalian necroptosis (23). The gene adjacent to *hellf* encodes an NLR with an NB-ARC domain and tetratricopeptide repeats (TPRs) termed FNT1 (Fig. 1B), which bears an N-terminal R0 region homologous to the HELLF R1 and R2 PFD repeats. HELLF behaves analogously to HET-S in every aspect that was analyzed. The C-terminal region of HELLF forms a prion termed [Φ], and [Φ] is incompatible with full-length HELLF. Upon interaction with [Φ], HELLF relocates to the cell membrane region and causes cell death. The N-terminal region of FNT1 forms fibrils and is able to induce [Φ] prion formation. The FNT1/HELLF system constitutes a second cell death-inducing amyloid signaling pathway in Podospora anserina. Solid-state nuclear magnetic resonance (NMR) revealed that HET-s and HELLF PFD amyloids have almost identical backbone structures in spite of extensive sequence divergence. It is noteworthy that, in spite of their almost identical backbone structure, [Het-s] and [Φ] prions do not cross-seed, thus indicating that NWD2/HET-S and FNT1/HELLF constitute two parallel signaling pathways.

In bioinformatics surveys, additional amyloid signaling motifs were identified in other fungi (15, 17). In particular, an amyloid motif termed PP was identified in the genome of *Chaetomium globosum* in a three-gene cluster (Fig. 1B). These genes were then functionally studied using heterologous expression in Podospora anserina. The NLR termed PNT1 regulates activation of two distinct effector proteins, an HeLo-like domain cell death-inducing protein termed HELLP and a putative lipase, SBP (renamed here CgPNT1, CgHELLP, and CgSBP for clarity). The PP motif is related in sequence to the RIP homotypic interaction motif (RHIM) amyloid motif that controls assembly of the RIP1 and RIP3 kinases in the necroptosis pathway in mammals (14, 23, 24). RHIM-like amyloid motifs also occur in the PGRP-LC, PGRP-LE, and Imd proteins controlling antibacterial immunity in Drosophila (25). The structure of the mammalian RIP1-RIP3 core (forming a heteroamyloid signaling complex) has recently been established (26). RHIM amyloids comprise two protein units per cross-β layer that run antiparallel and are interdigitated in a compact hydrophobic interface formed by the core G-(I,V)-Q-(I,V)-G motif. This central core motif is common to the PP motif (Fig. 1C), but in the absence of structural characterization of PP amyloids, it cannot at present be ascertained that this short sequence homology reflects structural similarity between RHIM and PP amyloids.

Here, we mine the genome of *P. anserina* and identify HELLP, a novel PP motif protein that defines, in that species, a third cell death-inducing amyloid signaling pathway comprising the HELLP cell death execution protein and an NLR (PNT1) (Fig. 1B). We show that the C-terminal PP motif of HELLP [HELLP(214-271)] forms a prion termed [π] in Podospora anserina and assembles into fibrils *in vitro.* We find that, like HET-S and HELLF, cell death-inducing activity of HELLP is activated by [π] prions formed by HELLP(214-271) or by the N-terminal region of PNT1, PNT1(1-31). We analyze in Podospora anserina the functional interactions of the HET-s, HELLF and HELLP PFD prions and find that these amyloid motifs control three independent signaling systems. We further analyze prion formation by human RHIMs from RIP1 and RIP3 kinases and find that [Rhim] and [π] prions partially cross-seed, underlining the functional similarity between these mammalian and fungal amyloid motifs.

## RESULTS

### A PP motif gene cluster in Podospora anserina.

During the course of a survey of NLR-encoding genes in fungi, we identified in Podospora anserina a gene encoding an NLR with an NB-ARC domain and TPRs (*Pa_5_8060*, PNT1), (15). We found after manual reannotation that the adjacent gene (*Pa_5_8070*) encodes an HeLo-like domain protein we termed HELLP (Fig. 1B). HELLP shows 56% similarity to the CgHELLP cell death execution protein of *Chaetomium globosum* (Fig. 1C; see also Fig. S1 in the supplemental material) (23). As previously reported for CgHELLP and HELLF, the HeLo-like domain of HELLP shows homology to the 4HB membrane-targeting domain of the mammalian MLKL necroptosis execution protein (Fig. S1). The N-terminal region of PNT1 and the C-terminal region of HELLP share a region of homology encompassing a predicted PP amyloid motif (Fig. 1C) (23). This region is also homologous to the RHIM found in the RIP1 and RIP3 kinases in humans (24, 26). Based on the resemblance with the PP gene cluster of C. globosum, we reasoned that after *nwd2*-*het-s* and *fnt1*-*hellf*, the *pnt1*-*hellp* gene pair might encode the components of a third amyloid NLR signalosome in *Podospora* (Fig. 1B). We thus engaged into the functional characterization of this gene pair.

10.1128/mBio.02782-20.5 FIG S1 Homology between HeLo or HELL domains of Podospora anserina and 4HB domain of human MLKL. Alignment of the HET-S HeLo domain and the HELLF and HELLP HELL domains of *P. anserina* with the 4HB region of the human MLKL. Alignment was generated with Clustal Omega with default settings. Download FIG S1, TIF file, 2.5 MB. Copyright © 2021 Bardin et al. 2021 Bardin et al. This content is distributed under the terms of the Creative Commons Attribution 4.0 International license.

### The C-terminal region of HELLP is a prion-forming domain.

**FIG 2:**
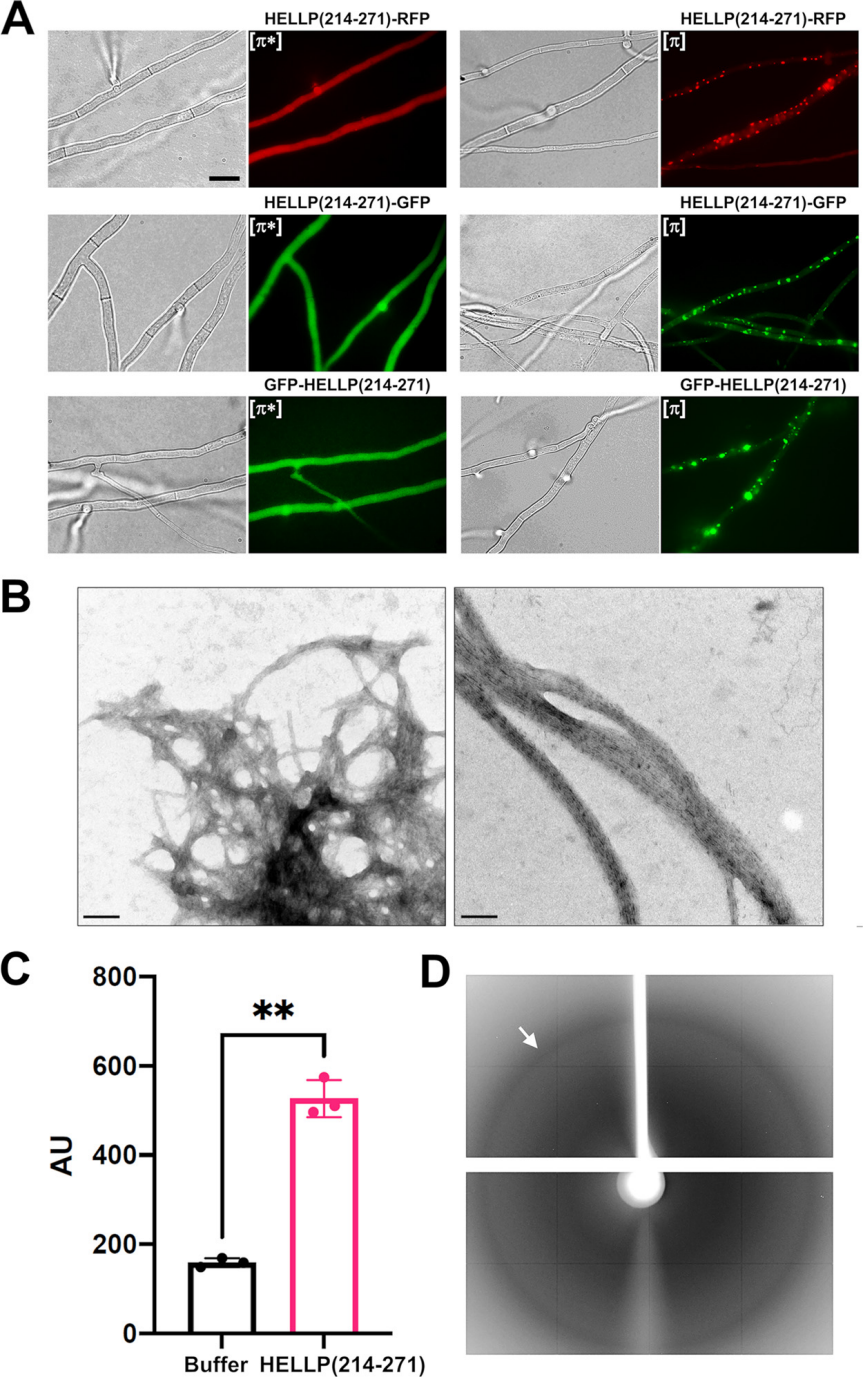
Characterization of the PP motif of *P. anserina.* (A) PP motif region of HELLP behaves as a prion-forming domain *in vivo*, as shown on micrographs of *P. anserina* strains expressing different molecular fusions [HELLP(214-271-RFP)/GFP and GFP-HELLP(214-271)], as indicated above each micrograph. Bar, 5 μm. Transformants initially present a diffuse fluorescence in the nonprion state designated [π*] (left) and systematically acquire dot-like fluorescent aggregates after contact with a strain already expressing the infectious prion state designated [π] (right). (B) The PP motif forms fibrils *in vitro*, as shown on an electron micrograph of negatively stained HELLP(214-271) fibrils (bar, 100 nm). (C) ThT-fluorescence signal of HELLP(214-271) fibrils. HELLP(214-271) fibrils and a buffer control were incubated with ThT, and fluorescence was measured (excitation wavelength, 440 nm; emission wavelength, 480 nm). **, *P* = 0.0028 (Welch’s test). (D) The X-ray diffraction pattern of unoriented HELLP(214-271) fibrils is given, and the reflection at 4.7 Å is marked by an arrow.

In order to analyze whether the C-terminal region of HELLP displays the anticipated prion-forming ability, we generated different constructs with the C-terminal domain of HELLP(214-271) fused either to green fluorescent protein (GFP) or red fluorescent protein (RFP) (in a C-terminal position) or to GFP in an N-terminal position. These constructs were expressed in the Δ*hellp* strain of *P. anserina*. For the different constructs, a population of 25 to 47 distinct transformants was analyzed by fluorescence microscopy. We observed either dot-like or diffuse fluorescence (Fig. 2A). The fraction of transformants exhibiting dot-like fluorescence was found to increase with growth duration (Table 1). The highest rate of foci formation was observed for the fusion protein bearing the GFP in an N-terminal position. Within 19 days, all tested GFP-HELLP(214-271) transformants displayed fluorescent foci. For all three fusion constructs, strains with diffuse fluorescence were systematically converted to the foci phenotype after cytoplasmic contact with strains expressing foci (Table 1), indicating that the foci state is transmitted by cytoplasmic contact and infectious. By analogy with the [Het-s] system, we term the phenotypic state with diffuse fluorescence [π*] and the foci state [π] (Fig. 2A). We conclude from these experiments that the PP motif region of HELLP(214-271) allows for prion formation and propagation. To determine whether, as previously described for [Het-s] (27), the [π] state could be reverted to [π*] in meiotic progeny, we analyzed 40 progeny of a *Δhellp Δhet-s Δhellf GFP-hellp(214-271)* [π] × *Δhellp Δhet-s Δhellf het-s-RFP* cross. Among the 20 progeny containing the *GFP-hellp(214-271)* transgene, three displayed a [π*] phenotype, indicating that, like [Het-s], the [π] prion can be cured in sexual crosses.

**TABLE 1:** Rates of spontaneous and induced prion formation and propagation^*a*^

Transgene^*b*^	Rate of spontaneous prion formation at no. of days after transfection:						Total no. monitored	Rate of induced prion formation	
	5		11		19				
	Diffuse (*n*)	Foci (*n* [%])	Diffuse (*n*)	Foci (*n* [%])	Diffuse (*n*)	Foci (*n* [%])		Diffuse (*n*)	Foci (*n* [%])
HELLP(214-271)-RFP	27	7 (21)	13	21 (62)	2	32 (94)	34	0	24 (100)
HELLP(214-271)-GFP	40	7 (15)	24	23 (49)	8	39 (83)	47	0	24 (100)
GFP-HELLP(214-271)	11	22 (67)	1	32 (97)	0	33 (100)	33	0	24 (100)
RIP3(444-469)-RFP	18	21 (54)	8	31 (80)	2	37 (95)	39	0	24 (100)
RIP3(444-469)-GFP	30	16 (35)	15	31 (67)	4	42 (91)	46	0	24 (100)
GFP-RIP3(524-551)	8	17 (68)	3	22 (88)	0	25 (100)	25	0	24 (100)
RIP1(524-551)-RFP	23	3 (12)	14	12 (46)	10	16 (62)	26	0	24 (100)
RIP1(524-551)-GFP	27	0 (0)	25	2 (7)	23	4 (15)	27	0	24 (100)
GFP-RIP1(524-551)	38	0 (0)	36	2 (5)	35	3 (8)	38	0	24 (100)
PNT1(1-31)-GFP	18	7 (28)			1	24 (96)	25	0	24 (100)
PNT1(1-31)-RFP	17	8 (32)			0	25 (100)	25	0	24 (100)

a For each transgene, between 25 to 47 different transformants were monitored by fluorescence microscopy for foci formation over 19 days. After 60 days, 100% of the transformants (except RIP1-GFP [33%] and GFP-RIP1 [16%]) displayed dots. At least two other transformation experiments were achieved for each transgene and generated 100% of tested transformants (*n* > 10) with foci in fluorescence after 60 days, except for RIP1-GFP (20%) and GFP-RIP1 (15%).

b RFP, red fluorescent protein; GFP, green fluorescent protein.

A larger construct with HELLP(171-271) fused to GFP either in the N or C terminus also led to foci formation (see Fig. S2A in the supplemental material), but in contrast to analogous HET-S and CgHELLP constructs [HET-S(157-289)-GFP and CgHELLP(170-278)-GFP], HELLP(171-271) fusions did not form elongated aggregates (23, 28).

10.1128/mBio.02782-20.6 FIG S2 [π] prion propagation of longer HELLP constructs. (A) Micrographs of *P. anserina* strains expressing HELLP(171-271)-GFP or GFP-HELLP(171-271), as indicated above each micrograph. Bar, 5 μm. Transformants presenting a diffuse fluorescence in the nonprion state designated [π*] (left) and dot-like fluorescence after contact with a [π] strain (right). (B) Barrage tests showing conversion of [π*] strains [expressing either HELLP(214-271)-GFP or HELLP(171-271)-GFP] to the [π] phenotype by contact with strains expressing GFP-HELLP(214-271) in the [π] state. Download FIG S2, TIF file, 2.4 MB. Copyright © 2021 Bardin et al. 2021 Bardin et al. This content is distributed under the terms of the Creative Commons Attribution 4.0 International license.

The HELLP(214-271) PFD region was also expressed in Escherichia coli with a C-terminal histidine tag and purified under denaturing conditions. Upon removal of the denaturant by dialysis, the protein spontaneously formed fibrils that often associated laterally as bundles (Fig. 2B). These fibrils induce ThT fluorescence (Fig. 2C). The ThT fluorescence signal was relatively modest, a situation previously observed for bacterial BASS3 fibrils (29) and HET-s amyloids (30). When analyzed by X-ray diffraction, HELLP fibrils showed the characteristic band at 4.7 Å typical of the cross-β arrangement (Fig. 2D). Based on these observations, we conclude that the HELLP(214-271) region forms amyloids *in vitro*, as previously observed for the CgHELLP PFD (23).

### [π] prions triggers incompatibility upon interaction with full-length HELLP.

**FIG 3:**
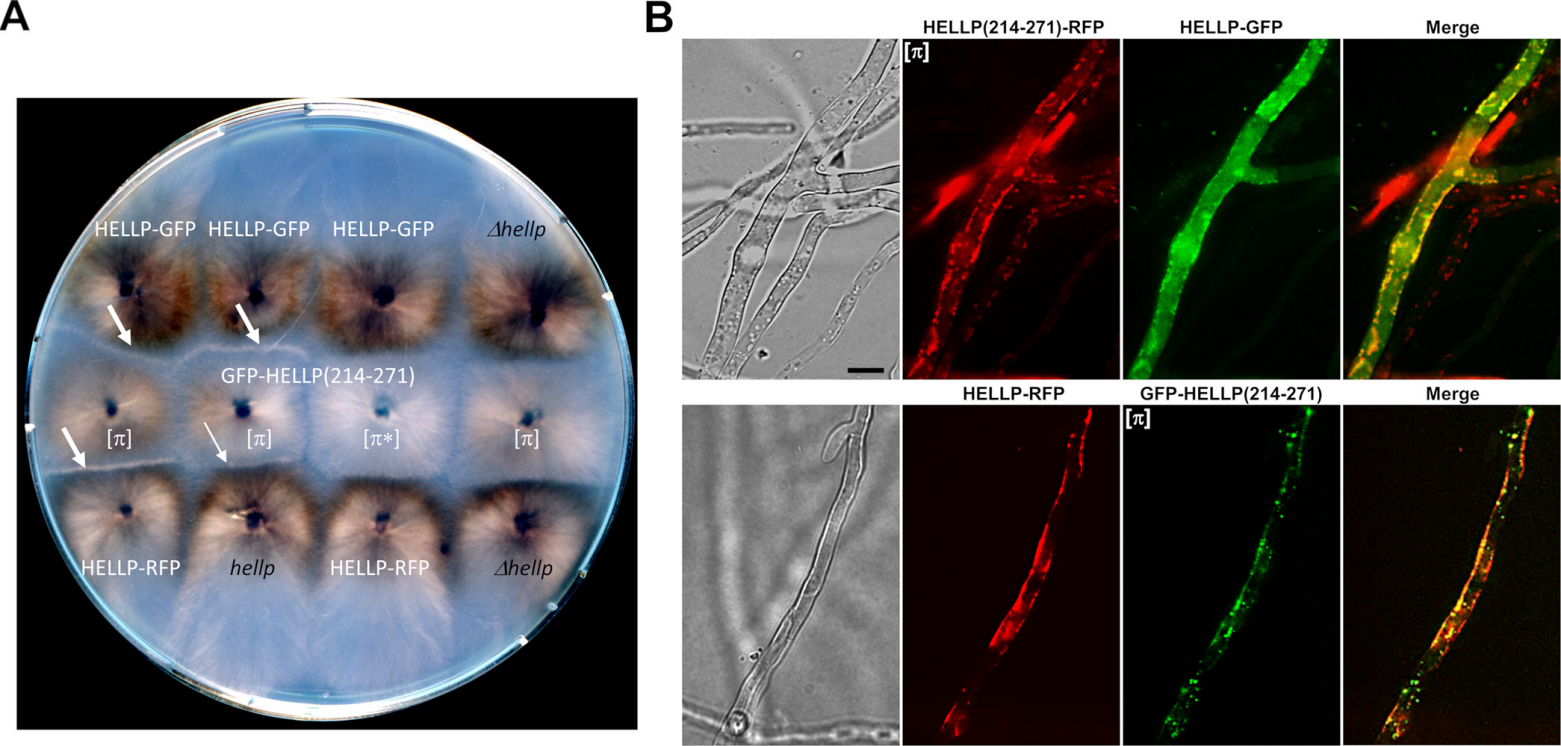
Activation of HELLP cell death-inducing activity by [π] prions. (A) Observation of incompatibility reaction (barrage) between a strain containing [π] prions and a strain expressing HELLP. Confrontation on solid medium of transformants expressing GFP-HELLP(214-271) in soluble [π*] or aggregated [π] states with strains expressing full-length HELLP from a transgene (labeled in white) or from the wild-type *hellp* gene or with the Δ*hellp* strain (both labeled in black). Barrages indicative of an incompatibility reaction are marked by white arrows. The thinner arrow indicates an attenuated barrage reaction. (B) Micrographs of fusion cells coexpressing full-length HELLP (HELLP-GFP [upper] or HELLP-RFP [lower panel]), and HELLP(214-271)-RFP (upper panel) or GFP-HELLP(214-271) (lower panel) in the [π] aggregated state. Note that HELLP relocalizes to the cell membrane region in the presence of [π]. Bar, 5 μm.

It was shown that HET-S, HELLF, and CgHELLP cell death-inducing activity is triggered by interaction with the prion form of their respective PFDs (7, 12, 21, 23). As a result, confrontation of strains expressing PFDs in the prion state with strains expressing the corresponding full-length protein leads to an incompatibility reaction that results in death of the fusion cells and, at the macroscopic level, in formation of an abnormal contact line termed “barrage” (21, 23, 27, 28). To determine whether HELLP could also cause an incompatibility reaction, strains expressing GFP-HELLP(214-271) were confronted with strains expressing full-length HELLP, either from the wild-type resident copy or from HELLP-GFP or HELLP-RFP transgene copies (in the Δ*hellp* background) (Fig. 3A). A barrage reaction was observed systematically and specifically in confrontations with strains expressing the [π] phenotype (as determined by presence of foci). [π*] strains did not produce a barrage reaction. A total of 24 transformants were analyzed in parallel for barrage formation and presence of foci, and the two phenotypes were strictly correlated. The barrage reaction was stronger with strains expressing HELLP-GFP or -RFP fusions from the strong *gpd* promoter than with strains expressing HELLP from the resident copy (Fig. 3A). Using the vital dye methylene blue, we verified that, at the microscopic level, barrage formation was indeed associated with cell death (see Fig. S3A in the supplemental material).

10.1128/mBio.02782-20.7 FIG S3 Death of fusion cells containing [π] or [Rhim] prions and HELLP full-length proteins. (A) Micrographs showing contact zone between HELLP-RFP and GFP-HELLP(214-271) (top)- or GFP-PNT1(1-31) (bottom)-expressing strains after methylene blue staining. Note that heterokaryotic fusion cells are stained (appeared in black), indicating their death. Bar, 2 μm. (B) Micrographs showing confrontations between HELLP-RFP and GFP-RIP3(444-469) (upper)- or GFP-RIP1(524-551) (lower)-expressing strains after methylene blue staining. Note that heterokaryotic fusion cells are stained, indicating their death. Bar, 2 μm. (C) Micrographs of strains expressing HELLP-GFP or HELLP-RFP fusion proteins. The fluorescence signal is diffuse and cytoplasmic and remains stable; cells are perfectly healthy. Bar, 5 μm. Download FIG S3, TIF file, 2.5 MB. Copyright © 2021 Bardin et al. 2021 Bardin et al. This content is distributed under the terms of the Creative Commons Attribution 4.0 International license.

As expected, the barrage formation phenotype was transmitted by cytoplasmic contact. Upon contact with barrage-forming strains, a compatible strain invariably became capable of forming a barrage with a strain expressing full-length HELLP (see Tables S1 to S4 and Fig. S2 in the supplemental material). Based on these experiments, the definition of the [π] phenotype can be expanded to represent both the foci state of the PP motif-GFP and -RFP fusions and the ability to produce a barrage reaction to full-length HELLP. The [π*]/[π] phenotypes of HELLP(214-271) are in that sense analogous to the [Het-s*]/[Het-s] phenotypes of HET-s (4) and the [Φ*]/[Φ] phenotypes of HELLF(209-277) (21).

10.1128/mBio.02782-20.1 Table S1 (A) [π] prion propagation assayed in barrage tests. (B) Test of cross-conversion between [π]^PNT1(1-31)^ and [π]^HELLP(214-271)^ prions. Download Table S1, DOCX file, 0.03 MB. Copyright © 2021 Bardin et al. 2021 Bardin et al. This content is distributed under the terms of the Creative Commons Attribution 4.0 International license.

It was shown that prion conversion of the HET-S PFD leads to the refolding of the HET-S HeLo domain that acquires pore-forming activity by exposing its N-terminal transmembrane helix, relocating it to the cell membrane and inducing cell death (7, 12). The same relocalization process was previously observed for HELLF and CgHELLP in the incompatibility reaction, leading to cell death (21, 23). We thus determined whether HELLP relocates in the cell membrane region upon interaction with [π] prions. We examined the contact zone between incompatible [π] and HELLP-GFP or HELLP-RFP-expressing strains (Fig. 3B). While full-length HELLP-GFP or HELLP-RFP alone yield a diffuse fluorescence signal that remains stable over time (Fig. S3C), in fusion cells coexpressing these proteins and the prion form of HELLP(214-271)-RFP or GFP-HELLP(214-271), we observed a relocalization of HELLP to the cell membrane region. We conclude that in these experiments, HELLP behaves as previously described for HET-S, HELLF, and CgHELLP and locates in the cell membrane region in fusion cells. Further experiments are required to determine whether HELLP directly interacts with the membrane and, if so, how this interaction causes cell death.

As previously described for HELLF and CgHELLP, a synthetic incompatibility system mimicking [Het-s]/HET-S can be derived from HELLP. As will be detailed in the discussion section, we do not infer from this that HELLP functions as a natural incompatibility gene in Podospora.

### The PNT1(1-31) region forms a prion and induces HELLP activation.

**FIG 4:**
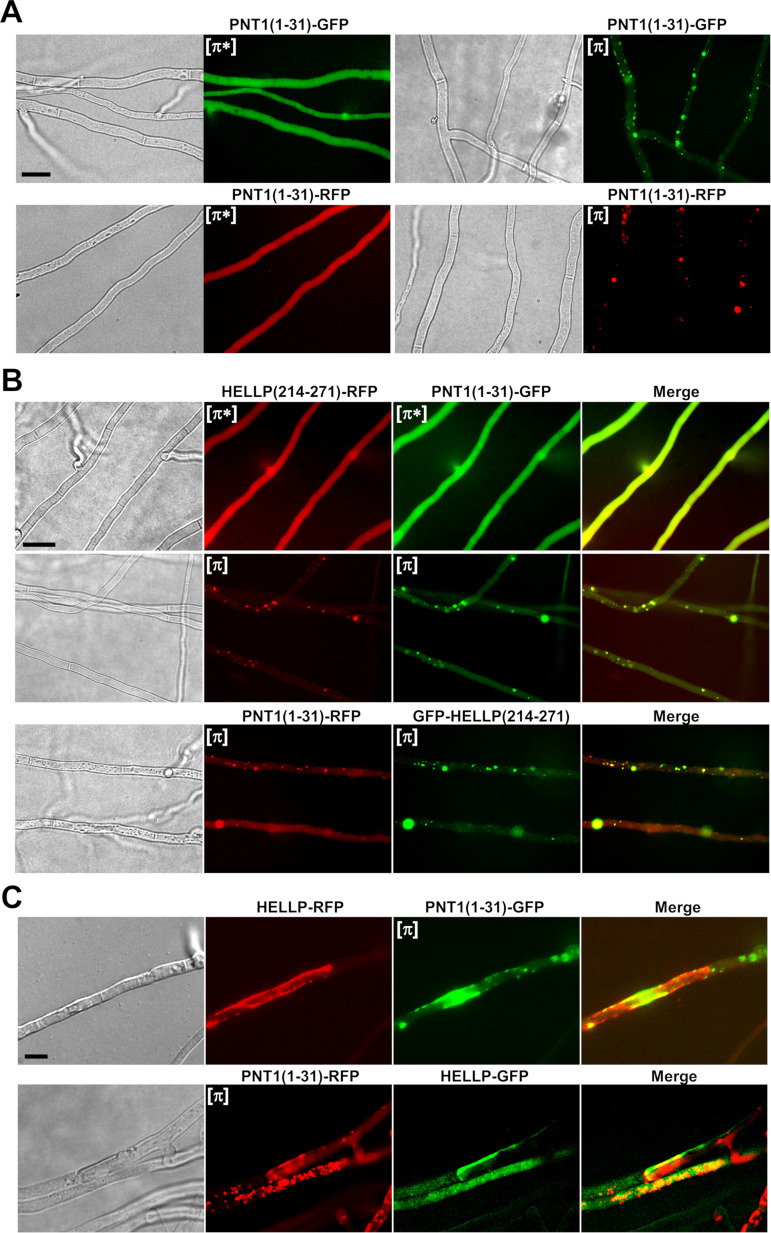
PNT1(1-31) forms [π] prions, colocalizes with HELLP(214-271), and induces toxicity of full-length HELLP. (A) Micrographs of PNT1(1-31) fusions with GFP or RFP in either the diffuse [π*] (left) or foci [π] state (right). (B) Micrographs showing colocalization of PNT1(1-31) and HELLP(214-271) in diffuse (top) or foci states. (C) In fusion cells coexpressing full-length HELLP (fused with RFP [upper] or GFP [lower]), and foci forms of PNT1(1-31) (fused with GFP [upper] or RFP [lower]), HELLP relocalized to the plasma membrane region. Bar, 2 μm.

It has been shown that the N-terminal region of NWD2 adopts an amyloid fold, displays prion infectivity, and is able to activate HET-S pore-forming activity (14). Similarly, the N-terminal regions of the FNT1 and CgPNT1 NLRs behave as PFDs and trigger cell death-inducing activity of their respective cognate effector proteins, HELLF and CgHELLP (21, 23). To determine whether the N-terminal region of PNT1 displays similar properties, we expressed PNT1(1-31) fused to GFP or RFP in Δ*hellp* strains. Two phenotypic states were observed, a diffuse cytoplasmic fluorescence state, and a foci state analogous to the [π*] and [π] phenotypes observed previously with HELLP(214-271) (Fig. 4A). Spontaneous conversion to the foci form occurred upon prolonged subculture (Table 1) and cytoplasmic contact with foci-containing strain-induced systematic conversion to the foci state (Table 1). PNT1(1-31)-GFP strains with fluorescent foci produced a barrage reaction to strains expressing full-length HELLP (data not shown). PNT1(1-31)-GFP [π] strains systematically convert [π*] HELLP(214-271)-RFP strains to the [π] state. Conversely, [π] HELLP(214-271)-RFP systematically converts [π*] PNT1(1-31)-GFP to the [π] state (Table S1B).

PNT1(1-31) and HELLP(214-271) were then coexpressed in the same strain (Fig. 4B). Fluorescence was either diffuse for both HELLP(214-271)-RFP and PNT1(1-31)-GFP or concomitantly dot-like for the two proteins. Cells in which one of the proteins formed foci while the other remained diffuse were not observed (Fig. 4B and Table S4A). HELLP(214-271)-RFP and PNT1(1-31)-GFP dots generally colocalized (Fig. 4B and Table S4B). The same was true during coexpression of PNT1(1-31)-RFP and GFP-HELLP(214-271). An initial diffuse state could not, however, be observed in this setting, presumably as the result of the high spontaneous conversion rate of the GFP-HELLP(214-271) fusion protein (Table 1).

Finally, we observed fusion cells between strains expressing PNT1(1-31) in the [π] state and strains expressing full-length HELLP (Fig. 4C and Fig. S3A). We observed the relocalization of HELLP to the plasma membrane region and cell death of the fusion cells. Thus, consistent with the proposed role of HELLP as cell death execution protein activated by the PNT1 NLR, we find that the PP motif region of PNT1 forms a prion and is able to activate HELLP.

### HET-S, HELLF, and HELLP are components of three independent cell-death inducing pathways.

We recently reported the lack of cross-seeding between [Het-s] and [Φ] prions, indicating the existence of two independent parallel amyloid signaling pathways in *P. anserina* (21). Here, we identify HELLP as an effector in a third amyloid signaling pathway in *P. anserina* and therefore wanted to analyze potential cross-interactions between these pathways. We thus analyzed [π*] conversion by [Het-s] and [Φ] prions (Table S2A). Strains expressing HELLP(214-271)-GFP or HELLP(214-271)-RFP and displaying the [π*] phenotype were confronted with strains expressing [π], [Φ], or [Het-s] prions and then tested for their ability to form a barrage with tester strains expressing full-length HELLP. Barrages were observed only (and systematically) when the prion donor strain expressed [π] prions, indicating that [π*] conversion does not occur in the presence of [Het-s] or [Φ] prions.

10.1128/mBio.02782-20.2 Table S2 (A)Assay in barrage tests of [π*] conversion by [Het-s] and [Φ] prions. (B) Test of cross-induction of incompatibility between three *P. anserina* amyloid signaling systems. Download Table S2, DOCX file, 0.03 MB. Copyright © 2021 Bardin et al. 2021 Bardin et al. This content is distributed under the terms of the Creative Commons Attribution 4.0 International license.

We also analyzed the cross-activation of the cell death effectors of the three systems by the different prions (Table S2B). We used incompatibility assays and confronted [π], [Φ], or [Het-s] prion-expressing strains with strains expressing either HELLP, HELLF, or HET-S full-length proteins. We observed barrage formation solely when [π] strains were confronted with HELLP-expressing strains, [Φ] with HELLF and [Het-s] with HET-S; that is, uniquely when the prion and the effector bear the same PFD. We observe no cross-interaction between these three amyloid signaling pathways.

**FIG 5:**
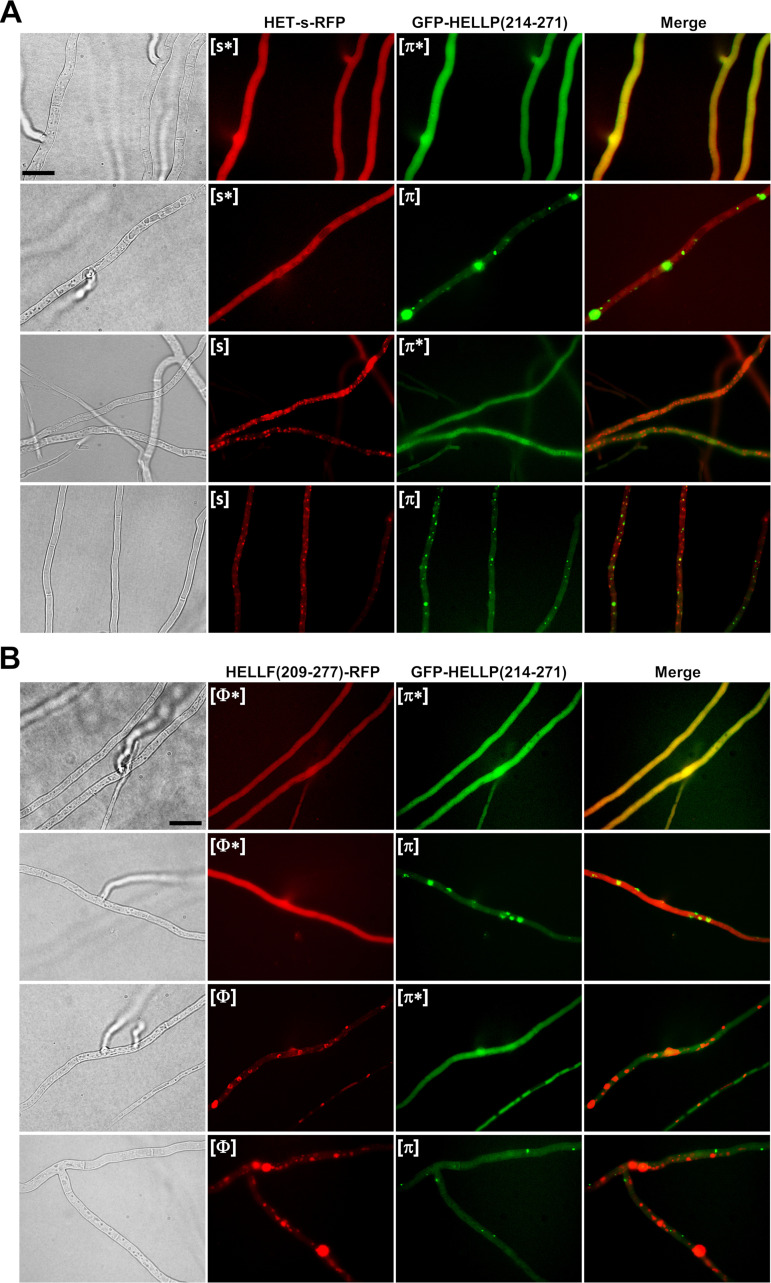
Lack of colocalization *in vivo* between [π] and [Het-s] or [Φ] prions. (A) Micrographs of strains coexpressing HET-s-RFP ([Het-s] or [Het-s*] states, noted here as [s*] and [s]) and GFP-HELLP(214-271) ([π*] or [π] state). Note an absence of colocalization of the prion forms and the independent occurrence of [π] and [Het-s]. Bar, 5 μm. (B) Micrographs of strains coexpressing HELLF(209-277)-RFP ([Φ] or [Φ*] state) and GFP-HELLP(214-271) ([π*] or [π] state). Note an absence of colocalization of the prion forms and the independent occurrence of [π] and [Φ] (bar, 5 μm).

To further analyze HELLP/HELLF and HELLP/HET-s PFD interactions, we coexpressed HELLP(214-271) with either HET-s or HELLF(209-271) in the Δ*hellp* Δ*het-s* Δ*hellf* strain. We observed the diffuse and foci states coexisting in all possible combinations (Fig. 5; see also Fig. S4 and Table S4A in the supplemental material). Namely, strains expressing HELLP(214-271) and HET-s could display four different phenotypes: [Het-s]/[π] and [Het-s*]/[π*] but also [Het-s]/[π*] and [Het-s*]/[π]. The same was true for HELLP(214-271) and HELLF(209-277) coexpressions (Fig. 5, Fig. S4, and Table S4A). These results are consistent with the prion conversion experiments and show that [Φ] or [Het-s] prions do not efficiently convert [π*] and, conversely, that [π] prions do not efficiently convert [Φ*] or [Het-s*]. We also noted an absence of colocalization between HELLP(214-271) and HET-s or HELLP(214-271) and HELLF(209-277) prion forms (Fig. 5 and Table S4B).

10.1128/mBio.02782-20.8 FIG S4 Absence of colocalization of HELLP(214-271)-RFP and HELLF(209-277)-GFP foci. Micrographs of strains coexpressing HELLP(214-271)-RFP ([π*] or [π] state) and HELLF(209-277)-GFP Φ([Φ*] or [Φ] state). Note that HELLP(214-271)-RFP and HELLF(209-277)-GFP foci do not colocalize ([Φ] and [π]), as well as the existence of cells in which one of the fusion protein forms foci while the other remains diffuse. Bar, 5 μm. Download FIG S4, TIF file, 2.4 MB. Copyright © 2021 Bardin et al. 2021 Bardin et al. This content is distributed under the terms of the Creative Commons Attribution 4.0 International license.

### *P. anserina* and *C. globosum* [π] prions cross-seed and colocalize.

It was shown previously that the HET-S-homologs from *P. anserina* and Fusarium graminearum, which share 37% identity in the PFD region, do cross-seed (31). We wondered whether the 36% identity between the PP-regions of HELLP and CgHELLP would also allow for cross-seeding (as suggested by the fact that pairwise identities between the different PP motifs within the *C. globosum* PP gene cluster or within the *P. anserina* PP gene pair are in the range of 32 to 48%, Fig. 1C).

**FIG 6:**
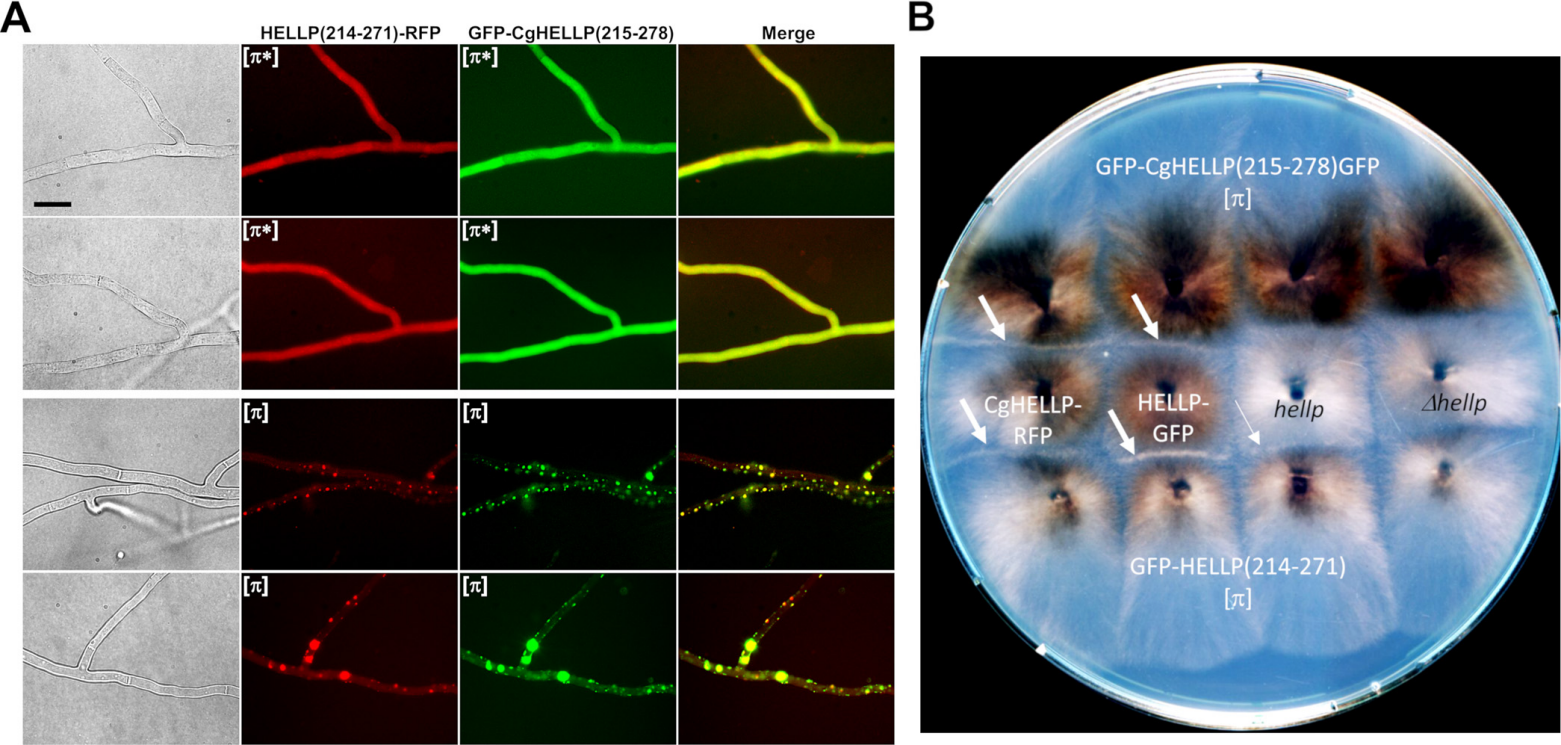
Colocalization of HELLP and CgHELLP PFDs and cross-induction of cell death activity. (A) Micrographs of strains coexpressing HELLP(214-271)-RFP and GFP-CgHELLP(215-278) in diffuse [π*] state (upper panels) or foci [π] state (lower panels). Note the colocalization of the two prion forms. Bar, 5 μm. (B) Confrontation on solid medium of strains expressing GFP-HELLP(214-271) and GFP-CgHELLP(215-278) [π] prions with strains expressing full-length HELLP from a transgene (marked in white) or from the wild-type *hellp* gene or with the Δ*hellp* strain (both marked in black). Barrages indicative of an incompatibility reaction are marked by a white arrow. The thinner arrow indicates an attenuated barrage reaction.

Upon confrontation, [π] GFP-HELLP(214-271) donor strains invariably converted GFP-CgHELLP(215-278) [π*] recipient strains and, conversely, [π] CgHELLP(215-278) strains converted GFP-HELLP(214-271) [π*] recipients (Table S3A). We analyzed the cross-induction of cell death activity of the full-length CgHELLP and HELLP proteins in barrage tests (Fig. 6B and Table S3A and B). GFP-CgHELLP(215-278) [π] strains produced a barrage reaction to strains expressing HELLP and, conversely, GFP-HELLP(214-271) [π] strains produced a barrage reaction to strains expressing CgHELLP. However, this was true only when HELLP was highly expressed as a transgene. No barrages were observed in confrontations to the wild type. This result suggests that heterologous activation of HELLP by GFP-CgHELLP(215-278) is less efficient than homotypic activation by GFP-HELLP(214-271).

10.1128/mBio.02782-20.3 Table S3 (A) Test of cross-conversion between [π]^Pa^ and [π]^Cg^ prions and induction of HELLP and CgHELLP toxicity. (B) Cross-induction of HELLP and CgHELLP cell death activity by [π] prions. Download Table S3, DOCX file, 0.03 MB. Copyright © 2021 Bardin et al. 2021 Bardin et al. This content is distributed under the terms of the Creative Commons Attribution 4.0 International license.

10.1128/mBio.02782-20.4 Table S4 (A) Occurrence of the different phenotypes observed using fluorescence microscopy during coexpression experiments. (B) Count of colocalized foci observed by fluorescence microscopy during coexpression experiments. Download Table S4, DOCX file, 0.03 MB. Copyright © 2021 Bardin et al. 2021 Bardin et al. This content is distributed under the terms of the Creative Commons Attribution 4.0 International license.

To examine if the two PFD regions could colocalize *in vivo*, strains coexpressing HELLP(214-271)-RFP and GFP-CgHELLP(215-278) were obtained and analyzed by fluorescence microscopy (Fig. 6A and Table S4). We initially observed diffuse fluorescence corresponding to the [π*] state and after a few days, foci appeared concomitantly for the two proteins. There was no coexistence of diffuse and foci forms, suggesting cross-conversion of HELLP and CgHELLP PFDs (Table S4A). Moreover, we observed significant colocalization, indicating coaggregation of the two PFDs (Fig. 6A).

We conclude that, as previously reported for F. graminearum and *P. anserina* HET-S homologs, the “species barrier” for prion seeding can be crossed for the PP motifs of *P. anserina* and *C. globosum*. This result is consistent with the fact that the level of within-cluster sequence identity of the PP motifs is comparable to the identity between CgHELLP and HELLP PP motifs. Nevertheless, the absence of barrage reaction between strains expressing [π]^Cg^ prions and HELLP at the wild-type level suggests that homotypic or within-cluster interactions are more efficient to activate the cell death activity of the full-length protein.

### The RHIMs of human RIP1 and RIP3 kinases form prions in *P. anserina*.

**FIG 7:**
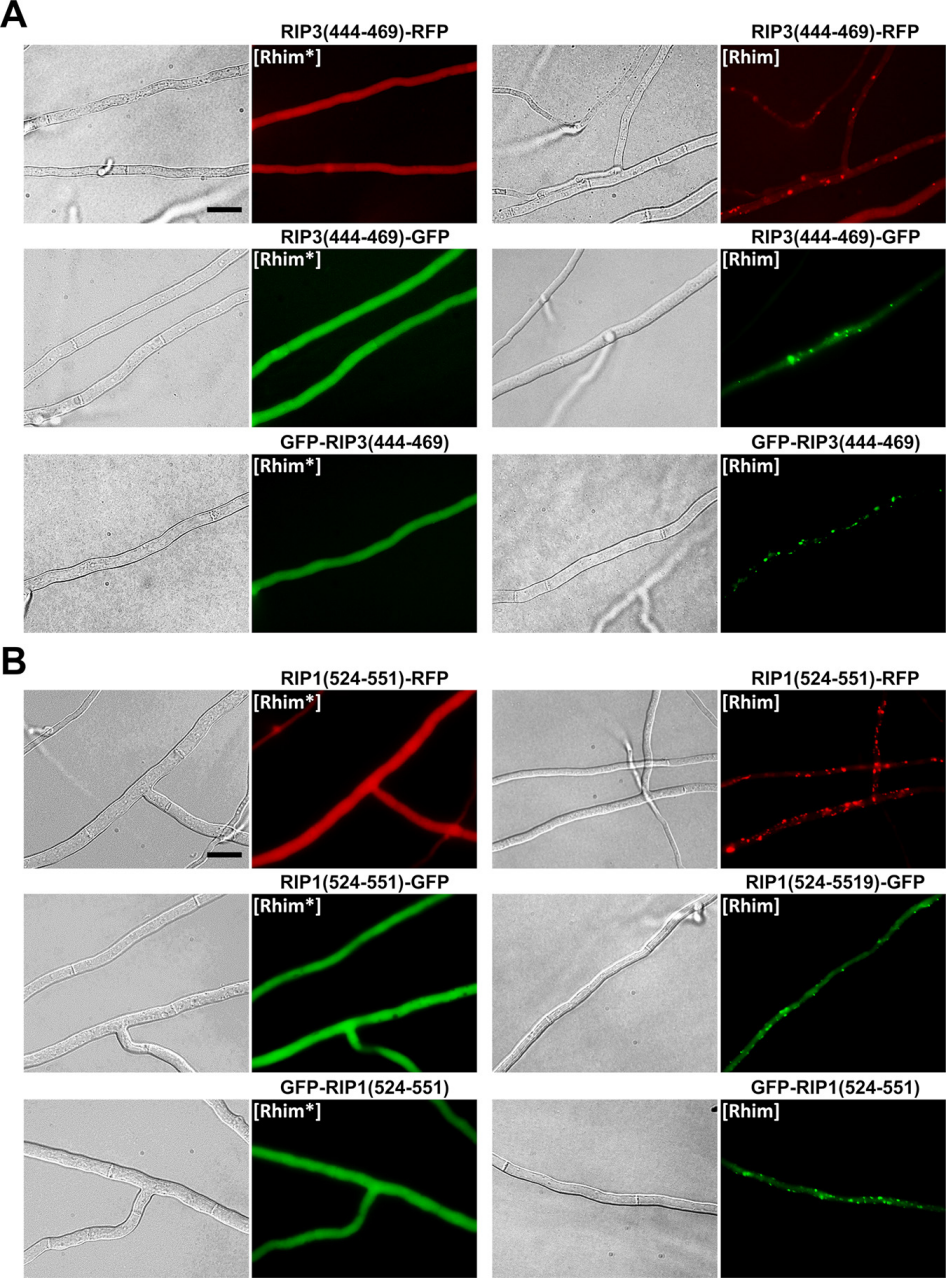
*In vivo* prion propagation of the human RIP3 and RIP1 RHIM regions in Podospora anserina. (A) Micrographs of *P. anserina* strains expressing the RIP3(444-469)-RFP, RIP3(444-469)-GFP, or GFP-RIP3(444-469) molecular fusions in the diffuse ([Rhim*]) and foci ([Rhim]) states. Bar, 5 μm. (B) Micrographs of *P. anserina* strains expressing the RIP1(524-551)-RFP, RIP1(524-551)-GFP, or GFP-RIP1(524-551) molecular fusions in the diffuse ([Rhim*]) and foci ([Rhim]) states. Bar, 5 μm.

The PP motif found in HELLP shows sequence homology to the mammalian RHIM amyloid motif (Fig. 1C). We thus envisioned that, like the PP motif, mammalian RHIMs might behave as PFDs when expressed in *Podospora*. We expressed the RIP1(524-551) and RIP3(444-469) regions fused to GFP or RFP in Δ*hellp* Δ*het-s* Δ*hellf* strains (Fig. 7). Initially fluorescence was diffuse and cytoplasmic, and foci appeared spontaneously after a few days of growth. The rate of spontaneous transition to the aggregated state was monitored as previously for HELLP (Table 1). For RIP3(444-469), the rates of foci formation were comparable to the those of PP motif, with the highest rate observed with GFP in an N-terminal position. For RIP1(524-551), the spontaneous rate of foci formation was lower, especially for the two GFP fusions. The induced conversion through cytoplasmic infection (with the corresponding foci form) was equally effective for the RIP1 and RIP3 RHIMs (Table 1). We designated the diffuse and infectious foci states [Rhim*] and [Rhim], respectively.

**FIG 8:**
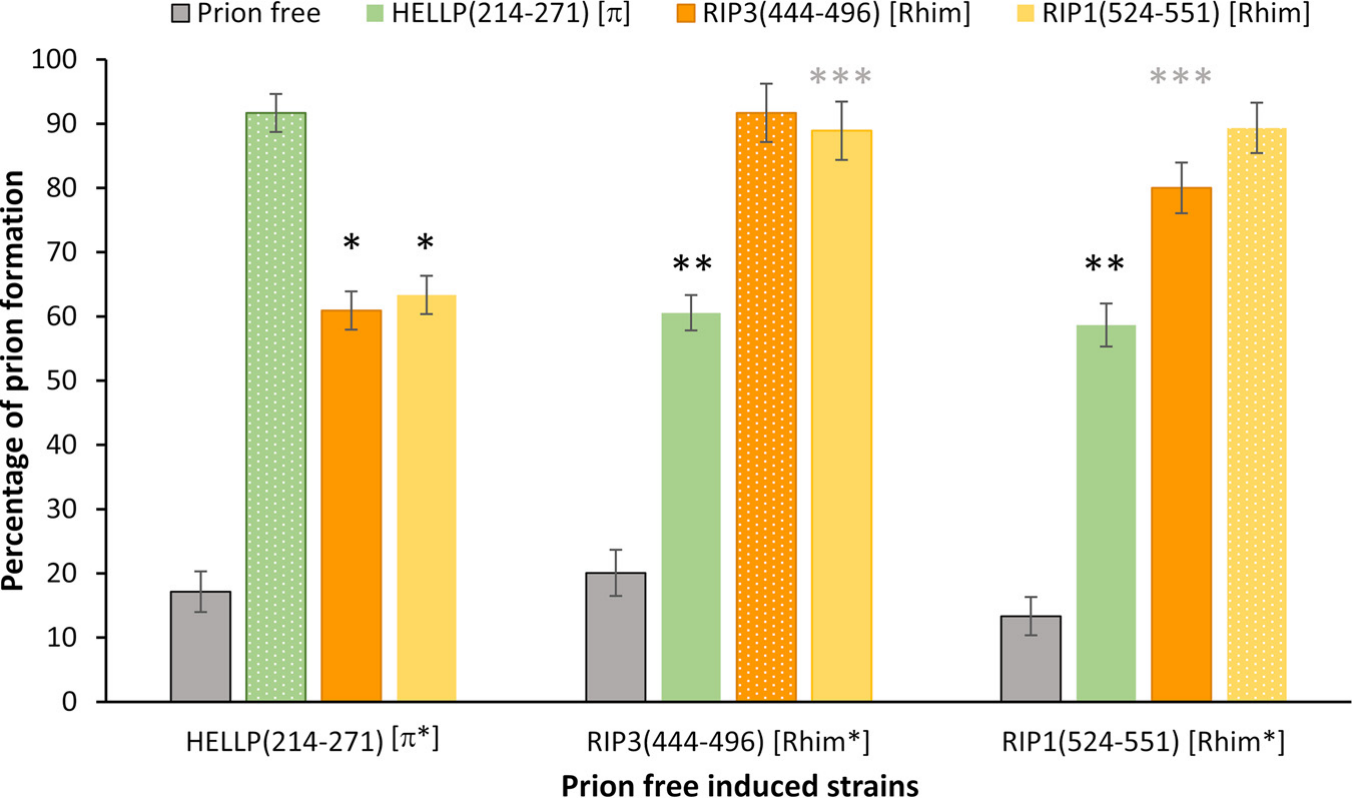
Cross-conversion between HELLP(214-271) [π] and [Rhim] prions. Histograms representing the percentage of [π] and [Rhim] prion-containing strains obtained after contact of recipient strains (initially displaying [π*] or [Rhim*] phenotype) with a Δ*hellp* Δ*het-s* Δ*hellf* prion-free strain (control), a GFP-HELLP(214-271) [π] strain, or GFP-RIP3(444-469) or GFP-RIP1(524-551) [Rhim] strains (as indicated on top). Phenotype after induction was determined by monitoring the acquisition of foci by fluorescence microscopy. Percentages of prion formation were expressed as the mean value ± standard deviation on 4 distinct transformants for each genotype (resulting in 56 to 108 independent infections per genotype). Homotypic inductions are showed with small dote patterns. *P* values for cross conversion were determined using a two-tailed Fisher’s test by comparison of the number of prion-free and prion-containing strains obtained after induction by the prion-free control or by the heterotypic prion-containing strain. * and **, *P* < 10^−8^; ***, *P* < 10^−12^.

**FIG 9:**
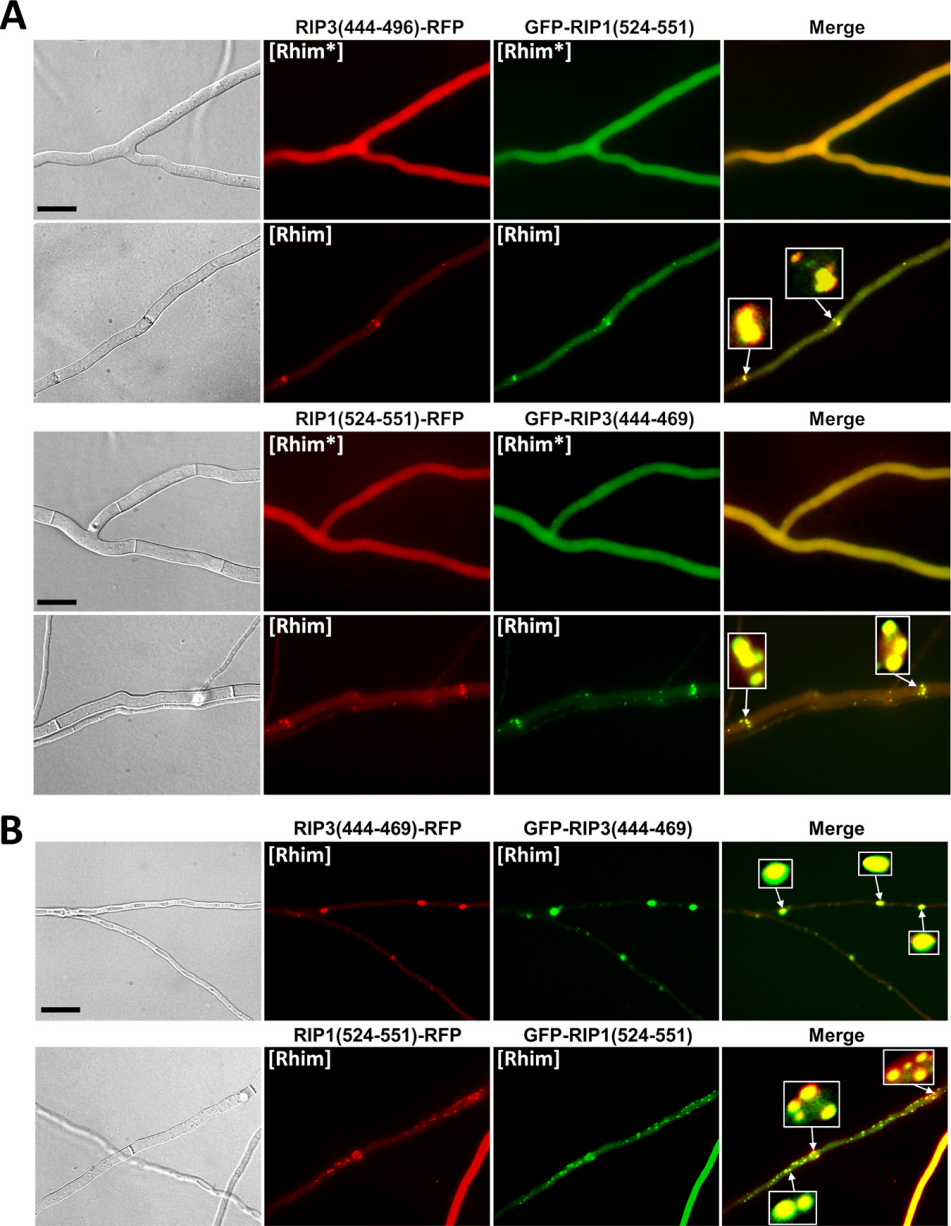
Colocalization of RIP1(524-551) and RIP3(444-469) foci in Podospora anserina. (A) Micrographs of *P. anserina* strains coexpressing the RIP3(444-469)-RFP and GFP-RIP1(524-551) or GFP-RIP3(444-469) and RIP1(524-551)-RFP molecular fusions. Bar, 5 μm. Note the strong colocalization between [Rhim] foci and dot alignment close to the septa (zoomed regions). (B) Micrographs of strains coexpressing GFP-RIP3(444-469) and RIP3(444-469)-RFP or GFP-RIP1(524-551) and RIP1(524-551)-RFP of [Rhim] phenotypes. Note the almost complete overlapping of foci of [Rhim] homopolymers. Bar, 5 μm.

Strains expressing RIP1(524-551) foci induced formation of RIP3(444-469) foci and the converse was also true (Fig. 8), showing cross-conversions between [Rhim]^RIP1^ and [Rhim]^RIP3^ prions. In these assays, heterotypic cross-induction rates between [Rhim]^RIP1^ and [Rhim]^RIP3^ were not significantly different from homotypic induction rates. To further analyze the interaction of [Rhim] prions propagated in *P. anserina*, we coexpressed RIP1(524-551) and RIP3(444-469) fused to GFP or RFP in the same strain (Fig. 9 and Table S4). Strains coexpressing RIP3(444-469) fused with GFP and RFP or RIP1(524-551) fused with GFP and RFP were used as positive controls (Fig. 9B) and showed colocalization with overlapping foci. In RIP1(524-551) and RIP3(444-469) coexpression experiments, diffuse fluorescence was observed initially. Then, foci formed concomitantly for both proteins. We observed no situation in which one of the proteins formed foci while the other remained diffuse, as previously observed for heterotypic PP motif interactions (Fig. 9 and Table S4B). RIP1(524-551) and RIP3(444-469) foci colocalized in the cell (Fig. 9 and Table S4B), often in the close vicinity of the septa, where they appeared as aligned dots.

We conclude that the RHIM containing RIP3(444-469) and RIP1(524-551) regions (of 26 and 28 amino acids in length, respectively) behave as PFDs *in vivo* in *P. anserina* and lead to the expression of two alternate phenotypes we termed [Rhim*] and [Rhim]. In addition, the RIP1 and RIP3 [Rhim] prions efficiently cross-seed and colocalize, consistent with the fact that in mammalian cells and *in vitro* RIP1 and RIP3 interact through their RHIM regions to form amyloid heteropolymers (26).

### The [Rhim] and [π] prions partially cross-seed.

Based on the similarity between PP motifs and RHIMs and on the fact that RIP1 and RIP3 RHIMs propagate as prions *in vivo* in *Podospora*, it was conceivable that [π] and [Rhim] prions might cross-seed to some extent. To examine possible interactions between [Rhim] and [π] prions, HELLP(214-271)-RFP [π*], GFP-RIP3(444-496) and GFP-RIP1(524-551) [Rhim*] strains were confronted with [π] and [Rhim] strains (Fig. 8). After 4 days, the recipient strains were sampled, subcultured, and analyzed for the presence of foci by fluorescence microscopy. We deliberately chose transformants expressing moderate levels of the fusion proteins to decrease the rates of spontaneous prion formation in these experiments. Under these test conditions, spontaneous prion formation was in the range of 10% to 20% for all constructs. Homotypic interactions led to 80% to 90% prion conversion (as mentioned above, high conversion rates were observed for heterotypic conversion of GFP-RIP3(444-496) [Rhim*] by GFP-RIP1(524-551) [Rhim] and GFP-RIP1(524-551) [Rhim*] by GFP-RIP3(444-496) [Rhim]). [π] prions induced formation of both [Rhim] prions at a rate of about 60%. Conversely, both [Rhim] prions induced formation of [π] prions at a similar rate (Fig. 8). These results indicate cross-conversion between [Rhim] and [π] prions. The conversion is, however, less efficient, as in the case of homotypic or intrakingdom [Rhim]^RIP1^/[Rhim]^RIP3^ or [π]^Pa^/[π]^Cg^ interactions.

We also tested the ability of the two [Rhim] prions to induce the conversion of [Het-s*] strains to the [Het-s] phenotype and observed cross-induction (18 transformants for each [Rhim] prion tested in triplicate). These results are in line with previous results indicating the absence of cross-interaction between [π] and the other amyloid signaling pathways of *P. anserina* and are thus rather expected.

**FIG 10:**
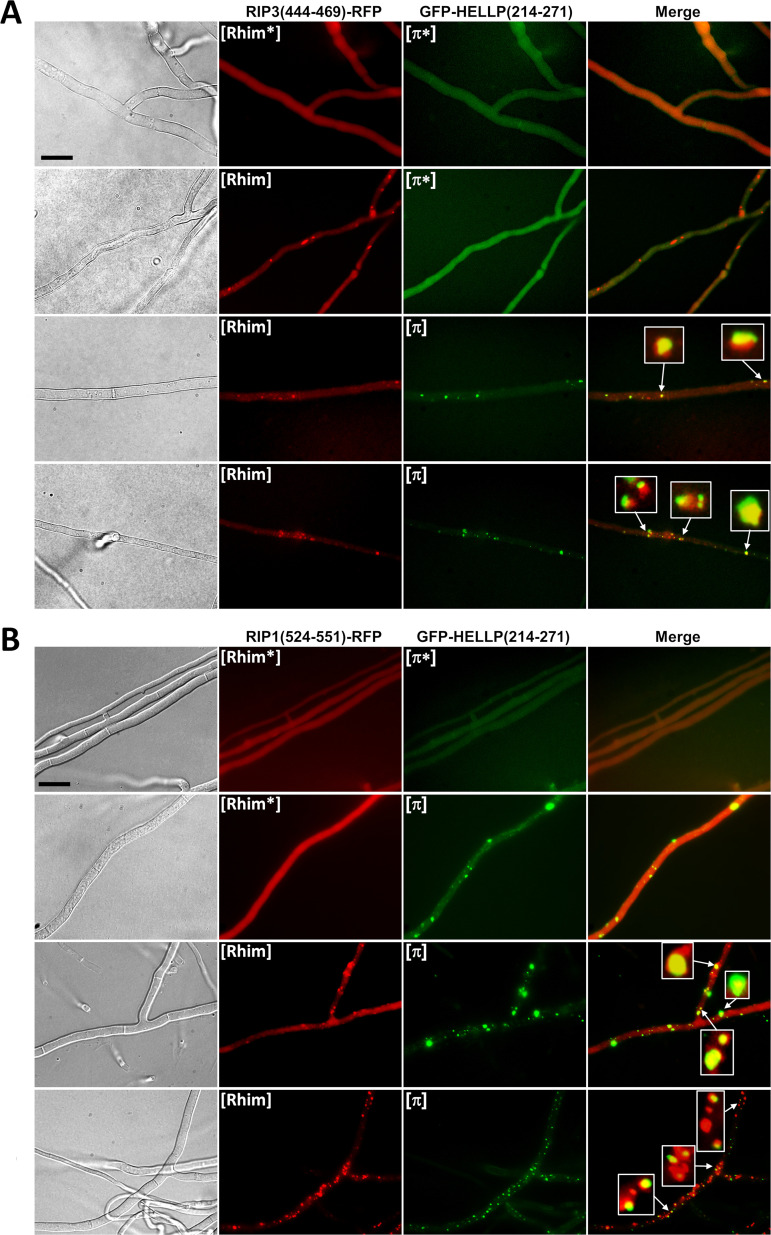
Partial colocalization of [π] and [Rhim] prions. (A) Micrographs of strains coexpressing RIP3(444-469)-RFP ([Rhim*] or [Rhim] states) and GFP-HELLP(214-271) ([π*] or [π] states). (B) Micrographs of strains coexpressing RIP1(524-551)-RFP ([Rhim*] or [Rhim] states) and GFP-HELLP(214-271) ([π*] or [π] states). In both cases, note the coexistence in the second lane of [Rhim] and [π*] (A) or [Rhim*] and [π] (B); these situations are only observable for a short period of time, and upon prolonged subculture, both prion forms occur together. Note also the partial colocalization of the two prions; some of the dots were zoomed to show incomplete overlapping in foci. Bar, 5 μm.

To explore further the interaction between [π] and [Rhim], HELLP and RIP3 (or RIP1) PFDs fused to GFP or RFP were coexpressed in the same strain, and transformants were analyzed by fluorescence microscopy (Fig. 10; see also Fig. S5 and Table S4 in the supplemental material). Consistent with the conversion experiments indicating partial cross-seeding, we could observe cells where one of the proteins formed foci while the other remain in the diffused state, as observed previously for non-cross-seeding prions (Fig. 10, Fig. S5, and Table S4A). In [π]/[Rhim] cells, the fraction of the foci that colocalized was higher (∼68%) than that between non-cross-seeding prions (such as HET-s and HELLP, for instance [∼5%]) but lower than in homotypic or [Rhim]^RIP1^/[Rhim]^RIP3^ or [π]^Pa^/[π]^Cg^ prions (∼82 to 96%). In addition, even in mixed foci the colocalization was imperfect, with RFP and GFP fluorescence that only partially overlapped (Fig. 10). This phenomenon of patchy colocalization has already been observed during coexpression of the HET-s proteins of F. graminearum and *P. anserina* (31). The same [π]/[Rhim] interaction experiments were also carried out with CgHELLP(215-278), and similar results were obtained (see Fig. S6 and Table S4 in the supplemental material). [π]^Cg^ and [Rhim] prions were found to partially cross-seed. In this case also, there was a partial colocalization of [Rhim] and [π] prions, a situation that is distinct both from the total lack of interaction seen between [π] and [Het-s] and [Φ] prions on the one hand and from the strong interactions seen between RIP1 and RIP3 [Rhim] prions or between HELLP and CgHELLP [π] prions on the other hand.

10.1128/mBio.02782-20.9 FIG S5 Partial colocalization of HELLP(214-271)-RFP foci and GFP-RIP1(524-551) and GFP-RIP3(444-469) foci. (A) Micrographs of strains coexpressing GFP-RIP3(444-469) ([Rhim*] or [Rhim] states) and HELLP(214-271)-RFP ([π*] or [π] states). (B) Micrographs of strains coexpressing GFP-RIP1(524-551) ([Rhim*] or [Rhim] states) and HELLP(214-271)-RFP ([π*] or [π] states). In both cases, note the coexistence on the second lane of [Rhim] and [π*] (A) or [Rhim*] and [π] (B). Note the partial colocalization of the foci; some of the foci were magnified to show incomplete overlap of the fusion protein. Bar, 5 μm. Download FIG S5, TIF file, 2.7 MB. Copyright © 2021 Bardin et al. 2021 Bardin et al. This content is distributed under the terms of the Creative Commons Attribution 4.0 International license.

10.1128/mBio.02782-20.10 FIG S6 Interaction between the human RIP3 or RIP1 RHIM region and the CgHELLP PFD. (A) Induction of [Rhim] prion by cross conversion of [Rhim*] by [π]^Cg^ or [Rhim] prions. Histogram representing the percentage of [Rhim*] strains displaying the [Rhim] phenotype after contact with an Δ*hellp* Δ*het-s* Δ*hellf* prion-free strain (control), a GFP-CgHELLP(215-278) [π]-containing strain or a GFP-RIP3(444-469) or GFP-RIP1(524-551) [Rhim]-containing strain (indicated on top). Strain phenotype after induction was determined by monitoring the acquisition of dot-like aggregates by fluorescence microscopy. Homotypic interactions are shown with a small dot pattern. Percentages of prion formation were expressed as the mean value ± standard deviation for 3 to 5 independent experiments on 4 different transformants. *P* values for cross conversion were determined using a two-tailed Fisher’s test by comparison of the number of prion-free and prion-containing strains obtained after induction by the prion-free control or by the heterotypic prion-containing strain and are <10^−8^. (B) Partial colocalization of [π]^Cg^ and [Rhim] prions *in vivo*. Micrographs of strains coexpressing RIP3(444-469)-RFP or RIP1(524-551)-RFP ([Rhim*] or [Rhim] states) and GFP-CgHELLP(215-278) ([π*] and [π] states). Note the coexistence of [Rhim*] and [π]; this situation is only observable for a short period of time, showing a low conversion rate of [Rhim*] by the [π] prion. Note the partial colocalization of the two prions; some of the dots were zoomed to show incomplete overlapping. Bar, 5 μm. Download FIG S6, TIF file, 2.5 MB. Copyright © 2021 Bardin et al. 2021 Bardin et al. This content is distributed under the terms of the Creative Commons Attribution 4.0 International license.

**FIG 11:**
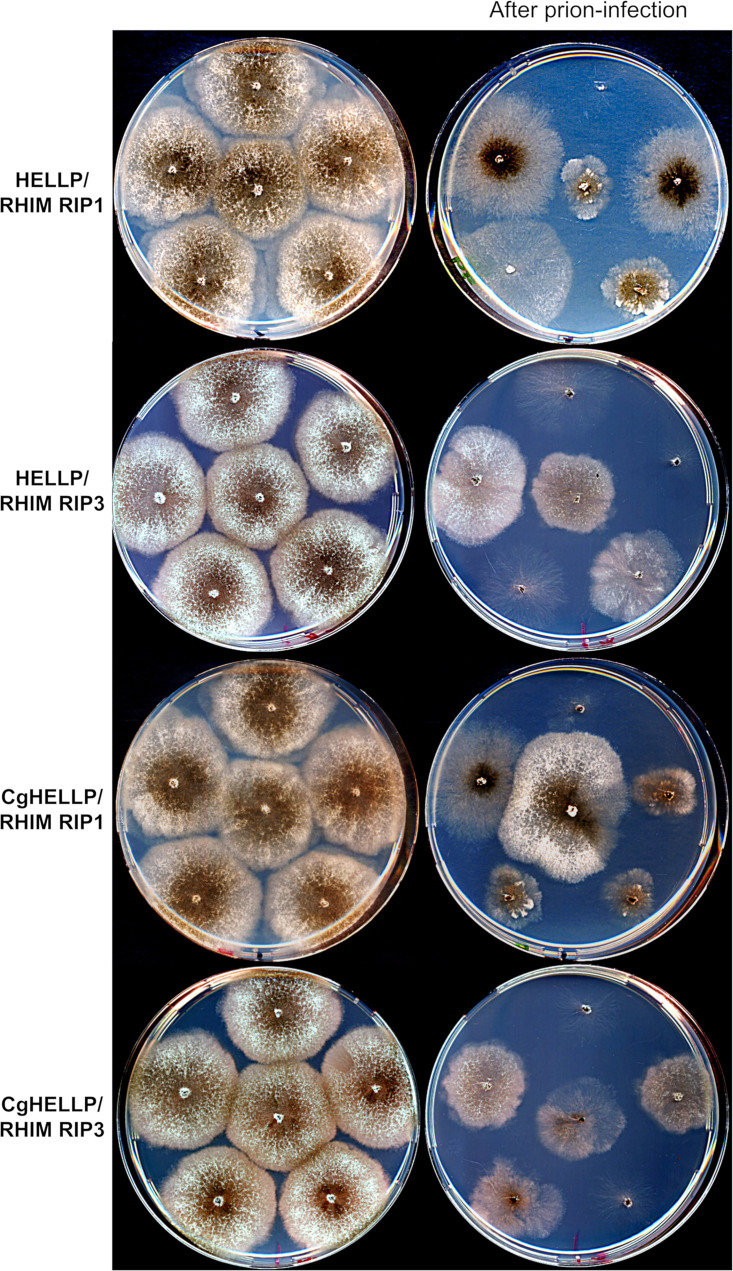
[Rhim] prions elicit self-incompatibility in strains coexpressing HELLP or CgHELLP and RIP1 or RIP3 RHIM. Comparison of growth on solid medium of strains coexpressing HELLP-RFP (HELLP) or CgHELLP-RFP (CgHELLP) and GFP-RIP1(524-551) (RIP1 RHIM) or GFP-RIP3(444-469) (RIP3 RHIM) before (left column) and after contact with a [Rhim] prion-containing strain (right column, after prion infection). Note that infection with [Rhim] prions leads to self-incompatibility, with growth alterations ranging from a sublethal phenotype to a more or less stunted growth.

The above [Rhim]/[π] cross-seeding and colocalization experiments suggest that RHIMs and PP motifs can interact but that this interaction is less efficient than that in PP homotypic pairings. We wondered whether [Rhim] prions could nonetheless induce, at least partially, HELLP or CgHELLP toxicity in a setting favoring [Rhim]/HELLP interactions. We thus coexpressed in the same strain GFP-RIP3(444-469) or GFP-RIP1(524-551) and HELLP-RFP or CgHELLP to determine whether prion conversion of [Rhim] in this strain would lead to growth alterations (self-incompatibility). Positive controls corresponding to cross-interacting incompatible combinations ([π]/HELLP) or negative controls corresponding to noninteracting combinations ([Φ] or [Het-s]/HELLP or [Rhim]/HET-S) were used for comparison (Table 2). In each combination, growth of a population of 12 to 39 transformants was observed before or after infection with the respective prion. Before prion infection, growth was normal for the vast majority of the transformants. After prion infection, a large fraction of the transformants (about 80%) coexpressing RHIM constructs and full-length HELLP or CgHELLP showed growth defects (Table 2 and Fig. 11). A similar proportion of growth defects was observed in known cross-interacting combinations, whereas growth alterations where not significantly increased in noninteracting combinations. The growth alteration phenotypes were highly heterogeneous (Table 2 and Fig. 11), ranging from sublethal growth to minor growth alteration. This phenotypic heterogeneity is typical of such self-incompatible situations and was also observed for the control self-incompatible strains. Phenotypic heterogeneity is thought to result from differences in transgene copy number and integration site in the different transformants and “escape” from self-incompatibility that is strongly selected for. Escape is manifested by the formation of growth sectors that recover close to normal growth and can occur by transgene mutation or deletion and from prion curing. Strains with [Rhim] prions do not lead to a barrage reaction when confronted with strains expressing full-length HELLP, suggesting that the RHIM/HELLP interaction is not efficient enough to induce a massive cell death reaction and barrage formation. However, we observed cell death in fusion cells between strains expressing HELLP-RFP and GFP-RIP3(444-469) or GFP-RIP1(524-551) [Rhim] strains (Fig. S3B). These results are consistent with the conversion experiments and indicate that [Rhim] prions are able to induce, to some extent, toxicity of HELLP (and CgHELLP), albeit at a lower efficiency than that of [π] prions.

**TABLE 2:** Growth alteration of strains coexpressing HELLP, CgHELLP, or HET-S cell death-inducing proteins and different prion-forming domains prior to and after prion infection

Coexpressed transgenes (PFD/cell death inducer)	No. of transformants analyzed	Growth before infection								Growth after infection							
		Normal		Altered		Sublethal		Total abnormal		Normal		Altered		Sublethal		Total abnormal	
		*n*	%	*n*	%	*n*	%	*n*	%	*n*	%	*n*	%	*n*	%	*n*	%
GFP-RIP3(444-469)/HELLP-RFP	15	14	93	1	7	0	0	1	7	3	20	9	60	3	20	12	80
RIP3(444-469)-RFP/HELLP-GFP	29	26	90	2	7	1	3	3	10	5	17	17	59	7	24	24	83
GFP-RIP3(444-469)/HET-S-RFP	18	16	89	1	6	1	6	2	11	15	83	2	11	1	6	3	17
RIP3(444-469)-RFP/HET-S-GFP	20	18	90	2	10	0	0	2	10	17	85	2	10	1	5	3	15
GFP-RIP1(524-551)/HELLP-RFP	36	31	86	3	8	2	6	5	14	7	19	20	56	9	25	29	81
RIP3(444-469)-RFP/HELLP-GFP	30	27	90	2	7	1	3	3	10	6	20	18	60	6	20	24	80
GFP-RIP1(524-551)/HET-S-RFP	18	16	89	1	6	1	6	2	11	15	83	3	17	0	0	3	17
RIP1(524-551)-RFP/HET-S-GFP	16	15	94	1	6	0	0	1	6	14	88	2	13	0	0	2	13
GFP(RIP3(444-459)/CgHELLP-RFP	39	35	90	4	10	0	0	4	10	6	15	22	56	11	28	33	85
GFP-RIP1(524-551)/HELLP-RFP	27	24	89	3	11	0	0	3	11	5	19	16	59	6	22	22	81
GFP-HELLP(214-271)/HELLP-GFP	18	15	83	2	11	1	6	3	17	3	17	10	56	5	28	15	83
HELLP(214-271)-RFP/HELLP-GFP	12	10	83	1	8	1	8	2	17	2	17	6	50	4	33	10	83
GFP-HELLF(209-277)/HELLP-RFP	34	31	91	1	3	1	3	2	6	28	82	3	9	3	9	6	18
HELLF(209-277)-RFP/HELLP-GFP	16	14	87	1	6	1	6	2	13	13	81	2	13	1	6	3	19
HET-s-RFP/HELLP-GFP	16	15	94	0	0	1	6	1	6	14	88	1	6	1	6	2	13

## DISCUSSION

A number of innate immune pathways function following a principle of signaling by cooperative assembly formation (SCAF) (32). Upon recognition of Pathogen-associated molecular patterns (PAMPs) or microbe-associated molecular patterns (MAMPs), receptors assemble into higher-order complexes or supramolecular organizing centers (SMOCs) (33). Following ligand recognition, the sensor modules oligomerize and recruit adaptor or effector proteins—often via homotypic interactions—that further amplify the oligomerization process and form cooperative, open-ended polymers (typically filaments). This general scheme applies to many immune signaling modules, including the RIG-I-like receptors and the AIM2-like receptors, and to NLRs. Formation of higher-order assemblies entitles such a signaling cascade with signal amplification, sharp threshold behavior, and noise reduction. This so-called prion-like polymerization process can involve nucleation of either folded globular domains such as the TIR or death domains (34, 35) or of short amyloid motifs such as RHIM identified in the necroptosis pathway in mammals. This motif allows for assembly of the necrosome formed by the RIP1 and RIP3 kinases (24). An RHIM-like motif also regulates amyloid formation in innate immune cascades in *Drosophila* (25). In addition, several NLR-associated amyloid signaling motifs were identified in filamentous fungi, and more recently in multicellular bacteria (19, 29). Among them is the PP motif that shows similarity to animal RHIMs and RHIM-like motifs (23). We identify here in the species Podospora anserina a PP motif two-component system comprising a NLR termed PNT1 and a cell death execution protein termed HELLP. As previously reported for CgHELLP from C. globosum, the N-terminal HeLo-like domain of HELLP shows homology to the 4HB domain of MLKL, which is responsible for membrane permeation in mammalian necroptosis. HELLP-induced RCD thus appears to be related to other evolutionarily widespread forms of immune RCD, which encompass fungal cell death, necroptosis, and cell death associated with the hypersensitive response in plants (23, 36, 37). We propose that PNT1 and HELLP constitute a third amyloid immune-related signaling cascade controlling cell fate in *P. anserina*. We show the absence of cross-induction between the three *P. anserina* systems, which allows for the coexistence of three independent amyloid signaling cascades in the same species. It is proposed that fungal NLRs akin to their plant and animal counterparts represent innate immune receptors that regulate response to pathogen and symbiotic interactions in fungi. The three amyloid-associated NLRs identified in *Podospora* share the fact that their WD or TPR ligand-binding domain is highly polymorphic and shows repeat length variation in natural populations consistent with its proposed role as an immune receptor (14, 15). Yet, except in the specific case of NLRs involved in incompatibility systems, the nature of the ligands activating these NLRs is currently unknown. Compared to the two other systems, NWD2/HET-S is characterized by the fact that this system has been exaptated as an allorecognition system in [Het-s]/HET-S incompatibility (13). It has been proposed that point mutations inactivating the HeLo domain converted HET-S into a naturally occurring prion. It has been possible to experimentally derive synthetic incompatibility systems analogous to [Het-s]/HET-S from both HELLP and HELLF. It will be of interest in the future to survey natural *Podospora* strains to determine whether exaptation of allorecognition incompatibility might have also occurred for HELLP and HELLF; that is whether derived prion forms of HELLP and HELLF altered in the HeLo-like domain exist in natural strains. It is also of note that the *Chaetomium globosum* and Podospora anserina PP-based signalosomes differ in the sense that the Chaetomium system has three components and also involves CgSBP, a lipase also comprising a PP motif and that is targeted to the membrane region by CgHELLP. The present study is superior to the previous study on the PP gene cluster in the sense that HELLP function is being studied here in the native context rather than by heterologous expression (22).

So far, four mammalian proteins involved in necroptosis and five *Drosophila* proteins also involved in immune signaling rely on RHIM-based amyloid signaling (24, 25). The facts that a range of viruses express RHIM-containing proteins and that pathogenic bacteria express an enzyme that specifically cleaves host RHIMs to prevent necrosome assembly highlight the crucial role of RHIM-based interactions in the host innate immune response (38). PP motifs present common features with other amyloid-forming domains, such as conservation of N/Q and G residues. N and Q allow for the formation of H-bonded N/Q ladders and G connect short adjacent β-strands (11, 39–41). PP motifs of CgHELLP and HELLP resemble RHIMs (Fig. 1B). This similarity occurs in the central G-ϕ-Q-ϕ-G core of the motif. The fungal PP motifs show strong conservation of the pseudopalindromic structure centered on the Q residue, along with conservation of flanking N and G residues. Mompeán and coworkers resolved the structure of the human RIP1-RIP3 amyloid region as a serpentine fold with turns and kinks resulting in an enclosed oblong-shaped hydrophobic core stabilized by N and Q ladders and Y stacking (26). If the sequence similarity between RHIM and PP reflect structural similarity, then it would appear that the PP fold is distinct from the HRAM β-solenoid fold of HET-s and HELLF. We find here that the RHIMs of RIP1 and RIP3 behave as PFDs when expressed in *Podospora* and thus behave analogously to PP motifs in that respect. The simple *in vivo Podospora* model designed here might represent a useful tool to study RHIM assembly and interactions, for instance, to screen for inducers or inhibitors of RIP1/RIP3 interactions.

Importantly, we find that [Rhim] and [π] prions cross-seed, albeit not as efficiently as RHIMs or fungal PP motifs. These results suggest both a structural similarity between RHIM and PP amyloids and substantial differences, since the efficiency of prion seeding is clearly distinct from that of homotypic or intraspecific seeding. Extensive structural analyses of the amyloid fibers of HELLP or CgHELLP will be required to determine to which extend PP-based amyloid polymers share the same fold as RIP1-RIP3. It appears, however, that the partial cross-seeding between PP and RHIM supports a model of a common evolutionary origin of the two motifs. In this context, it is interesting to note that, in addition to the RHIM-like motifs described in *Drosophila*, an RHIM-related amyloid motif termed BASS3 was also identified in multicellular bacteria (29). It might be that the RHIM-related motif represents an archetypal amyloid motif largely conserved through evolution, while other motifs such as HRAMs or other bacterial signaling motifs have a more restricted phylogenetic distribution.

## MATERIALS AND METHODS

### Gene annotation.

The Pa_5_8070 and Pa_5_8060 genes were annotated manually using two intron prediction programs, GENSCAN (hollywood.mit.edu/GENSCAN.html) and NetGene2 (www.cbs.dtu.dk/services/NetGene2/), and based on BLASTN searches against expressed sequence tag (EST) sequences at NCBI and transcriptome sequencing (RNA-Seq) data from reference 42. A single 47-bp intron was identified in nucleotide positions 429 to 475 of the Pa_5_8070 open reading frame (ORF); this newly defined ORF encodes a 271-amino-acid (aa) protein named HELLP. A single intron of 54 bp was identified in nucleotide positions 95 to 148 of the ORF of Pa_5_8060, TPR number in the 3′ part of the ORF is variable in *P. anserina* strains; we described here the ORF of the S Orsay reference strain, which encodes a 971-aa protein named PNT1.

### Strains and plasmids.

The *P. anserina* strains used in this study were wild-type *het-s* or *het-S* and the strains *Δhellp* (*ΔPa_5_8070*) *het-s°* (23) and *Δhellp (ΔPa_5_8070) Δhet-s (ΔPa_3_620) Δhellf (ΔPa_3_9900)*, obtained by crossing the *Δhet-s Δhellf* strain (21) with the *Δhellp het-s°* strain. The *Δhellp het-s°* strain was used as a recipient strain for the expression of molecular fusions of full-length HELLP or PP motif-containing regions of HELLP and the GFP (green fluorescent protein) or RFP (red fluorescent protein). These fusions were expressed from plasmids based on the pGEM-T backbone (Promega), named pOP plasmids (23) and containing either the GFP or RFP, or in a derivative of the pAN52.1 GFP vector (28), named the pGB6-GFP plasmid. In both cases, the molecular fusions were under the control of the strong constitutive *P. anserina* glyceraldehyde-3-phosphate dehydrogenase (*gpd*) promoter. The *Δhellp het-s°* strain was transformed as described previously (43) with a fusion construct, along with a second vector carrying the phleomycin-resistance gene *ble*, pPaBle (using a 10:1 molar ratio). Phleomycin-resistant transformants were selected, grown for 30 h at 26°C, and screened for the expression of the transgenes using fluorescence microscopy. The *hellp* gene and the *hellp(171-271)* and *hellp(214-271)* gene fragments were amplified with the respective 5′ forward oligonucleotides 5′-ggcttaattaaATGGATCCTCTCAGTATCACAGC-3′, 5′-ggcttaattaaATGATCACAGCGACAAACGATCAG-3′, and 5′-ggcttaattaaATGAAAGTACTGCATGAATCGCGC-3′, and the same 3′ reverse oligonucleotide, 5′-ggcagatcttgctccCCCCCTTCGGCCAAATGTAG-3′ (capital letters correspond to *P. anserina* genomic DNA sequences). The PCR products were cloned upstream of the GFP- or RFP-coding sequence in the pOP plasmids using PacI/BglII restriction enzymes to generate the pOPhellp-GFP, pOPhellp-RFP, pOPhellp(171-271)-GFP, pOPhellp(171-271)-RFP, pOPhellp(214-271)-GFP, and pOPhellp(214-271)-RFP vectors, in which, in addition to the BglII site, a 2-amino-acid linker (GA) was introduced between the sequences encoding HELLP and GFP or RFP. *hellp(171-271)* and *hellp(214-271)* were also amplified with the respective 5′ forward oligonucleotides 5′-ggcgcgcggccgcATCACAGCGACAAACGATCAG-3′ and 5′-ggcgcgcggccgcATCACAGCGACAAACGATCAG-3′ and the same 3′ reverse oligonucleotide, 5′-ggcggatccCTACCCCCTTCGGCCAAATG-3′, and cloned downstream of the GFP using NotI/BamHI restriction enzymes to generate the plasmids pGB6-GFP-hellp(171-271) and pGB6-GFP-hellp(214-271).

To investigate the colocalization of HELLP with CgHELLP or of HELLP with HELLF or HET-s, the *Δhellp Δhet-s Δhellf* strain was transformed both with vectors expressing fluorescently tagged versions of full-length or truncated HELLP described above and with fluorescently tagged versions of full-length or truncated HELLF, HET-s, HET-S, or CgHELLP as previously described (21, 23, 28), namely, pOPhellf-GFP, pOPhellf(209-277)-RFP, pOPhellf(209-277)-GFP, pOPhellf(L52K)-GFP, pOPhet-s-RFP, pGPD-het-S-GFP, pOPhet-S-RFP, and pGB6-GFP-Cghellp(215-278).

In the same way the sequence encoding the first 31 amino acids of *pnt1* (*Pa_5_8060*) was amplified with the oligonucleotides 5′-ggcttaattaaATGTCAGACAGTTATCGTTTCGGC-3′ and 5′-ggcagatcttgctccTGGCGCCTGCAGGAAAGTATTG3′ and cloned in the pOP plasmids using PacI/BglII restriction enzymes to generate the pOPpnt1(1-31)-GFP and the pOPpnt1(1-31)-RFP. The *Δhellp Δhet-s Δhellf* strain was transformed with these vectors (and pPaBle), either alone or with pOPhellp(214-271)-RFP or pGB6-GFP-hellp(214-271).

For heterologous expression in E. coli, we cloned *hellp(214-271)* in pET24 (Novagen) using the NdeI/XhoI restriction sites. The gene fragment *hellp(214-271)* was amplified with the oligonucleotides 5′-agccatatgAAAGTACTGCATGAATCGCGC3′ and 5′-agcctcgagCCCCCTTCGGCCAAATGTAG-3′ and cloned in front of a polyhistidine tag to generate the pET24-hellp(214-271)-6×His plasmid.

For the expression of RIP3(444-469) and RIP1(524-551), PCR products were amplified with 5′-ggcttaattaaATGGTTACCGGTCGTCCGCTG-3′ and 5′-ggcagatcttgctccCTGCATGGTCAGGTAGTTGTTG-3′ or 5′-ggcgcggccgcGTTACCGGTCGTCCGCTGG-3′ and 5′-ggcGGATCCTTACTGCATGGTCAGGTAGTTG-3′ for RIP3 and with 5′-ggcttaattaaATGACTGACGAATCCATCAAATACACC-3′ and 5′-ggcagatcttgctccGCCGCCGATTTCCATGTAGTT-3′ or 5′-ggcgcggccgcACTGACGAATCCATCAAATACACC-3′ and 5′-ggcGGATCCTTAGCCGCCGATTTCCATGTAGTT-3′ for RIP1 and cloned as described above in the pOP plasmids using PacI/BglII restriction enzymes or in pGB6-GFP plasmid using NotI/BamHI restriction enzymes.

### Microscopy.

P. anserina hyphae were inoculated on solid medium and cultivated for 24 to 72 h at 26°C. The medium was then cut out, placed on a glass slide, and examined with a Leica DMRXA microscope equipped with a Micromax charge-coupled device (CCD) (Princeton Instruments) controlled by MetaMorph 5.06 software (Roper Scientific). The microscope was fitted with a Leica PL APO 100× immersion lens. To observe cell death reactions and HELLP relocalization, HELLP-GFP- or HELLP-RFP-expressing strains were inoculated at a distance of 2 cm from a strain expressing one of the fusion proteins containing an HELLP PFD, PNT1(1-31), RIP3(444-469), or RIP1(524-551) fused to GFP or RFP. The confrontation zone between the two strains was observed 12 to 48 h after contact. For methylene blue staining, an aqueous 0.5% solution was put directly on the mycelium for 1 min, followed by washing with distilled water before observation as described above.

For HELLP fibril observations, negative staining was performed as follows. Aggregated proteins were adsorbed onto Formvar-coated copper grids (400 mesh) and allowed to dry for 15 min in air; grids were then negatively stained for 1 min with 10 μl of freshly prepared 2% uranyl acetate in water, dried with filter paper, and examined with a Hitachi H7650 transmission electron microscope (Hitachi, Krefeld, Germany) at an accelerating voltage of 120 kV. Transmission electron microscopy (TEM) was performed at the Pôle Imagerie Électronique of the Bordeaux Imaging Center using a Gatan USC1000 2k × 2k camera.

### Incompatibility assays (barrage tests).

Methods for determination of incompatibility phenotypes were previously described (21, 44). In brief, incompatibility phenotypes were determined by confronting strains on solid cornmeal agar medium, and a barrage reaction (abnormal contact lines forming upon confrontation of incompatible strains) was assessed 3 days postcontact. The [π] phenotype (acquisition of the [π] prion) was assessed as the ability of a strain to form a barrage with a wild-type strain or with a *Δhellp* strain bearing a transgene-encoding full-length HELLP in fusion with GFP or RFP, termed the “tester strain.” Cross-reaction between the different prion systems was also assessed in barrage tests as the ability of a prion-containing strain (either [π], [Rhim], [Het-s], or [Φ]) to form a barrage with a strain expressing one of the full-length HeLo- or HELL-containing proteins HELLP, CgHELLP, HELLF, or HET-S. In these experiments, strains previously described by Daskalov and colleagues were used (21, 23).

### Prion propagation.

Methods for determination of prion formation and propagation were previously described (21, 44). Prion formation and propagation can be observed either by using microscopy to monitor the apparition of dots or by using barrage tests to observe the formation of a barrage with the tester strain.

Spontaneous prion formation is first monitored as the rate of spontaneous acquired prion phenotype (dots) in the initially prion-free subculture after 5, 11, and 19 days of growth at 26°C on cornmeal agar using microscopy, as described. Then transformants were reobserved at least 60 days after transformation to confirm prion acquisition. For [π] strains, barrage tests were realized 5, 19, and after at least 60 days and a strict correlation between the presence of dots and barrage formation with HELLP-expressing strains was always observed.

Prion formation can also be measured as the ability to propagate prions from a donor strain (containing prion) to a prion-free strain (induced strain). In practice, prion-free strains are confronted on solid cornmeal agar medium for 4 to 6 days (this step is mentioned as “previous contact with” in the tables) before being subcultured and observed by fluorescence microscopy and analyzed in barrage tests. As indicated in the figure legends, at least 12 different transformants were used, and the tests were realized in triplicates. Again, for [π] strains, barrage and dot formation are strictly correlated. Prion acquisition through induction can also be visualized directly on solid medium by positioning the inocula of donor and prion-free strains closer than the tester strain (see Fig. S2 in the supplemental material) to allow prion propagation before contact with the tester strain.

It is of note that transformants were randomly tested for prion formation, allowing various expression levels of the transgene (high levels of expression are usually associated with very rapid spontaneous prion formation), except for the cross-conversion test (see Fig. 8 and Fig. S6 in the supplemental material), where transformants expressing moderate level of transgene were preferred to limit the rate of spontaneous transition within the timing of the experiment that could mask the prion induction. For this experiment, a statistical analysis was performed using a two-tailed Fisher’s test to determine *P* values and validate test results, as indicated in the figure legend.

### Protein preparation and fibril formation.

HELLP(214-271) protein was expressed in E. coli BL21-CodonPlus-RP competent cells as insoluble proteins and purified under denaturing conditions using its terminal 6-histidine tag as previously described (45). Briefly, cells were grown at 37°C in DYT medium (NaCl [5 g/liter], tryptone/peptone [16 g/liter], yeast extract [10 g/liter]) to an optical density at 600 nm (OD_600_) of 0.6, and expression was induced with 1 mM isopropyl β-d-1-thiogalactopyranoside. After 4 h, cells were harvested by centrifugation, frozen at −80°C, sonicated on ice in a lysis buffer (Tris 50 mM, 150 mM NaCl [pH 8]), and centrifuged for 20 min at 20,000 × *g* to remove E. coli contaminants. The pellet containing HELLP(214-271) in inclusion bodies was washed in the same buffer and resuspended in denaturing buffer (8 M guanidinium HCl, 150 mM NaCl, and 100 mM Tris-HCl [pH 8]) until complete solubilization. The lysate was incubated with Talon resin (Clontech) for 1 h at 20°C, and the resin was extensively washed with 8 M urea, 150 mM NaCl, and 100 mM Tris-HCl (pH 8). The protein was eluted from the resin in the same buffer containing 200 mM imidazole. The protein HELLP(214-271) was pure, as judged by sodium dodecyl sulfate polyacrylamide gel electrophoreses (SDS-PAGE) followed by Coomassie blue staining, and yield was in the range of ∼2 to 4 mg of protein per liter of culture. To eliminate urea, elution buffer was replaced by overnight dialysis at 4°C against Milli-Q water. Fibril formation resulted spontaneously from the dialysis process followed by sample storage at 4°C for 7 days. Other conditions were used for sample preparation to test the influence of salt or acidic buffer on fibril formation, namely, presence or absence of 500 mM NaCl in the dialysis buffer, or replacement of dialysis buffer by 50× dilution in Milli-Q water or in ammonium acetate buffer 100 mM (pH 4.5). All resulted in the spontaneous appearance of fibrils.

### ThT fluorescence assay.

The aggregated HELLP fibril samples supplied with 20 μM ThT were transferred to a 96-well Corning plate (transparent bottom) with 100 μl/well. The fluorescence measurements were carried out on three independent samples in a CLARIOstar Plus plate reader with 10 flashes per well (excitation wavelength, 440 nm; emission wavelength, 480 nm). Seven independent measurements are averaged and plotted.

### X-ray diffraction.

The fiber diffraction pattern was measured at 4°C on a Rigaku FRX rotating anode X-ray generator at the copper wavelength (Kα, λ = 1.54 Å). The source was equipped with Osmic Varimax HF optics and a Dectris Eiger 1M detector on a 2θ arm of a Rigaku partial chi AFC11 goniometer. The sample was mounted in a MicroLoop from MiTeGen on a goniometer head under the cold nitrogen flow. The diffraction pattern corresponds to a 360° rotation along the phi axis (perpendicular to the direct beam with omega and chi axes at the 0 position) with an exposure time of 720 sec. Data were integrated with CrysalisPro (Rigaku Oxford Diffraction, Ltd., Yarnton, Oxfordshire, England) with median filter and baseline correction.

### Bioinformatics methods.

BLAST analyses on the *P. anserina* genome were achieved on the Podospora anserina Genome Project site (podospora.i2bc.paris-saclay.fr/). Sequence alignments were performed with Clustal Omega or MAFFT (www.ebi.ac.uk) and edited with Jalview (www.jalview.org/). To generate the hidden Markov model (HMM) profile signatures for PP and RHIM, PSI-BLAST searches were performed (blast.ncbi.nlm.nih.gov) using the HELLP PP sequence and the RHIMs of human RIP3, matching sequences were aligned with Clustal Omega, and the alignment was analyzed with Skylign (skylign.org). Secondary structure predictions were performed with PSIPRED (bioinf.cs.ucl.ac.uk/psipred/). Hidden Markov model searches were performed using HHPred (toolkit.tuebingen.mpg.de) and Jackhhmer (www.ebi.ac.uk/Tools/hmmer/search/phmmer), both with default settings. The prediction of transmembrane helix was performed with the TMHMM server (www.cbs.dtu.dk/services/TMHMM).
