# Supplementary material for: Partial Prion Cross-Seeding between Fungal and Mammalian Amyloid Signaling Motifs
Source: mBio. 2021 Feb 9;12(1):e02782-20. doi: 10.1128/mBio.02782-20 (PMC7885112; doi:10.1128/mBio.02782-20)
Supplement: Table S1 [file mBio.02782-20-st001.docx]

**Table S1A. [π] prion propagation assayed in barrage tests.**

|  | |  |  | | | | |  | | tester strains | | | | |  | | |  |
| --- | --- | --- | --- | --- | --- | --- | --- | --- | --- | --- | --- | --- | --- | --- | --- | --- | --- | --- |
| tested strain  (prion recipient) | |  | previous contact with  (prion donor) | | | | |  | | *ΔhellpΔhet-sΔhellf* | | | | | recipient strain | | |  |
| recipient strain | transgene |  | | recipient strain | transgene | |  | | | HELLP-GFP | | HELLP-RFP | | transgene | | |  |  |
| *Δhellp*  *het-s°* | GFP-HELLP (214-271)  [π*] | | *Δhellp*  *het-s°* | | | no transgene | | |  | | 0/12 | | 0/12 | | |  | | |
|  |  |  |  |  |  | GFP-HELLP(214-271) [π] | | |  | | 12/12 | | 12/12 | | |  | | |
|  |  |  |  |  |  | HELLP (214-271)-GFP [π] | | |  | | 12/12 | | 12/12 | | |  | | |
|  |  |  |  |  |  | HELLP (214-271)-RFP [π*] | | |  | | 0/12 | | 0/12 | | |  | | |
|  |  |  |  |  |  | HELLP (214-271)-RFP [π] | | |  | | 12/12 | | 12/12 | | |  | | |
|  |  |  |  |  |  | HELLP (171-271)-GFP [π] | | |  | | 12/12 | | 12/12 | | |  | | |
|  |  | |  | | | GFP-HELLP (171-271) [π] | | |  | | 12/12 | | 12/12 | | |  | | |

**Table S1B. Test of cross-conversion between [π]^PNT1(1-31)^ and [π]^HELLP(214-271)^ prions**

|  | |  |  |  | | |  | tester strains | | | |  | |
| --- | --- | --- | --- | --- | --- | --- | --- | --- | --- | --- | --- | --- | --- |
| tested strain  (prion recipient) | | |  | previous contact with  (prion donor) | | |  | *ΔhellpΔhet-sΔhellf* | | | | recipient strain | |
| recipient strain | transgene | |  | recipient strain | | transgene | HELLP-RFP | | HELLP-GFP | | transgene | |  |
| *Δhellp*  *Δhet-sΔhellf* | HELLP  (214-271)-RFP  [π*] | | *Δhellp*  *Δhet-sΔhellf* | | PNT1(1-31)-GFP  [π] | | | 12/12 | | 12/12 | |  | |
|  |  |  |  |  | PNT1(1-31)-GFP  [π*] | | | 0/12 | | 0/12 | |  | |
|  |  |  |  |  | PNT1(1-31)-RFP  [π] | | | 12/12 | | 12/12 | |  | |
|  |  |  |  |  | PNT1(1-31)-RFP  [π*] | | | 0/12 | | 0/12 | |  | |
| *Δhellp Δhet-sΔhellf* | PNT1(1-31)-RFP  [π*] | | *Δhellp Δhet-sΔhellf* | | GFP-HELLP (214-271)  [π] | | | 12/12 | | 12/12 | |  | |

The tables S1A and B gives the number of transformants producing a barrage reaction (after contact with the given prion donor strain), to two different tester strains expressing full-length HELLP (either as GFP or RFP fusion). For each transgene, 12 different transformants were tested and the experiment were done in triplicate. All triplicates were consistent.
