# Supplementary material for: Partial Prion Cross-Seeding between Fungal and Mammalian Amyloid Signaling Motifs
Source: mBio. 2021 Feb 9;12(1):e02782-20. doi: 10.1128/mBio.02782-20 (PMC7885112; doi:10.1128/mBio.02782-20)
Supplement: Table S2 [file mBio.02782-20-st002.docx]

**Table S2A. Assay in barrage tests of [π*] conversion by [Het-s] and [Φ] prions**

|  | |  |  |  | | |  | tester strains | | |  |  |
| --- | --- | --- | --- | --- | --- | --- | --- | --- | --- | --- | --- | --- |
| tested strain  (prion recipient) | | |  | previous contact with  (prion donor) | | |  | *ΔhellpΔhet-sΔhellf* | | | recipient strain |  |
| recipient strain | transgene | |  | recipient strain | | transgene |  | HELLP-RFP | | HELLP-GFP | transgene | |
| *Δhellp*  *Δhet-sΔhellf* | HELLP  (214-271)-GFP  [π*] | | *Δhellp*  *Δhet-sΔhellf* | | HET-s-RFP  [Het-s] | | | 0/12 | 0/12 | |  |  |
|  |  |  |  |  | GFP-HELLF(209-277)  [Φ] | | | 0/12 | 0/12 | |  |  |
|  |  |  |  |  | GFP-HELLP (214-271)  [π] | | | 12/12 | 12/12 | |  |  |
| *Δhellp*  *Δhet-sΔhellf* | HELLP  (214-271)-RFP  [π*] | | *Δhellp*  *Δhet-sΔhellf* | | HET-s-RFP  [Het-s] | | | 0/12 | 0/12 | |  |  |
|  |  |  |  |  | GFP-HELLF(209-277)  [Φ] | | | 0/12 | 0/12 | |  |  |
|  |  |  |  |  | GFP-HELLP (214-271)  [π] | | | 12/12 | 12/12 | |  |  |

The table gives the number of transformants producing a barrage reaction (after contact with the given prion donor strain), to two different tester strains expressing full-length HELLP (either as GFP or RFP fusion). For each transgene, 12 different transformants were tested and the experiment were done in triplicate. All triplicates were consistent.

**Table S2B. Test of cross-induction of incompatibility between three *P. anserina* amyloid signaling systems**

|  |  |  | tester strain | | |  |
| --- | --- | --- | --- | --- | --- | --- |
| tested strains | |  | *ΔhellpΔhetsΔhellf* | | | recipient strain |
| recipient strain | transgene |  | HET-S-GFP | HELLF-GFP | HELLP-GFP | transgene |
| *ΔhellpΔhet-sΔhellf* | HET-s-RFP | | 18/18 | 0/18 | 0/18 |  |
| *Δhet-s* | HET-s(218-289)-GFP | | 18/18* | 0/18 | 0/18 |  |
| *ΔhellpΔhet-sΔhellf* | HELLF(209-277)-RFP | | 0/18 | 18/18 | 0/18 |  |
| *ΔhellpΔhet-sΔhellf* | GFP-HELLF(209-277) | | 0/18 | 18/18 | 0/18 |  |
| *ΔhellpΔhet-sΔhellf* | HELLF(L52K)-GFP | | 0/18 | 18/18 | 0/18 |  |
| *ΔhellpΔhet-sΔhellf* | HELLP(214-271)-GFP | | 0/18 | 0/18 | 18/18 |  |
| *ΔhellpΔhet-sΔhellf* | GFP-HELLP(214-271) | | 0/18 | 0/18 | 18/18 |  |
| *Δhellp het-s°* | GFP-CgHELLP(215-278) | | 0/18 | 0/18 | 18/18 |  |

The table gives the number of transformants producing a barrage reaction to the given tester strains. For each transgene, 18 different transformants were tested and the experiment was done in triplicate. All triplicate were consistent. *As noted previously, HET-s(218-289)-GFP produces an attenuated barrage reaction to HET-S.
