# Supplementary material for: Partial Prion Cross-Seeding between Fungal and Mammalian Amyloid Signaling Motifs
Source: mBio. 2021 Feb 9;12(1):e02782-20. doi: 10.1128/mBio.02782-20 (PMC7885112; doi:10.1128/mBio.02782-20)
Supplement: Table S3 [file mBio.02782-20-st003.docx]

**Table S3A. Test of cross-conversion between [π]^Pa^ and [π]^Cg^ prions and induction HELLP and CgHELLP toxicity**

|  | |  |  | |  | |  | tester strains | |  |
| --- | --- | --- | --- | --- | --- | --- | --- | --- | --- | --- |
| tested strain  (prion recipient) | | |  | previous contact with  (prion donor) | | |  | *Δhellp*  *het-s°* | *Δhellp*  *Δhet-sΔhellf* | recipient strain |
| recipient strain | transgene | |  | recipient strain | | transgene |  | CgHELLP-RFP | HELLP-RFP | transgene |
| *Δhellp*  *Δhet-sΔhellf* | GFP-HELLP  (214-271)  [π*] | | *Δhellp*  *Δhet-sΔhellf* | | | none | | 0/12 | 0/12 |  |
|  |  |  | *Δhellp*  *Δhet-sΔhellf* | | | GFP-HELLP (214-271)  [π] | | 12/12 | 12/12 |  |
|  |  |  | *Δhellp*  *het-s°* | | | GFP-CgHELLP (215-278)  [π] | | 12/12 | 12/12 |  |
| *Δhellp*  *het-s°* | GFP-CgHELLP  (215-278)  [π*] | | *Δhellp*  *Δhet-sΔhellf* | | | none | | 0/12 | 0/12 |  |
|  |  |  | *Δhellp*  *Δhet-sΔhellf* | | | GFP-HELLP (214-271)  [π] | | 12/12 | 12/12 |  |
|  |  |  | *Δhellp*  *het-s°* | | | GFP-CgHELLP (215-278)  [π] | | 12/12 | 12/12 |  |

The table gives the number of transformants producing a barrage reaction (after contact with the given prion donor strain), to two different tester strains expressing either full-length HELLP-RFP or full-length CgHELLP-RFP. For each transgene, 12 different transformants were tested and the experiment done in triplicate. All triplicates were consistent.

**Table S3B. Cross-induction of HELLP and CgHELLP cell death activity by [π] prions.**

|  | |  | tester strains | | | | |  |
| --- | --- | --- | --- | --- | --- | --- | --- | --- |
| tested strains | |  | *Δhellp*  *Δhet-sΔhellf* | *Δhellp*  *Δhet-sΔhellf* | *Δhellp*  *Δhet-sΔhellf* | *wt* | *Δhellp het-s°* | recipient strain |
| Recipient strain | Transgene |  | none | HELLP-RFP | HELLP-GFP | none | CgHELLP-RFP | transgene |
| *Δhellp*  *Δhet-sΔhellf* | none | | 0/18 | 0/18 | 0/18 | 0/18 | 0/18 |  |
| *Δhellp*  *Δhet-sΔhellf* | GFP-HELLP (214-271) | | 0/18 | 18/18 | 18/18 | 18/18^a^ | 18/18 |  |
| *Δhellp*  *Δhet-sΔhellf* | GFP-HELLP (171-271) | | 0/18 | 18/18 | 18/18 | 18/18^a^ | 18/18 |  |
| *Δhellp*  *Δhet-sΔhellf* | HELLP  (214-271)-RFP | | 0/18 | 18/18 | 18/18 | 18/18^a^ | 18/18 |  |
| *Δhellp het-s°* | GFP-CgHELLP (215-278) | | 0/18 | 18/18 | 18/18 | 0/18 | 18/18 |  |
| *Δhellp het-s°* | CgHELLP  (170-278)-RFP | | 0/18 | 18/18 | 18/18 | 0/18 | 18/18 |  |

The table gives the number of transformants producing a barrage reaction to the given tester strains (**^a^** barrage was attenuated). For each transgene, 18 different transformants were tested and the experiment was done in triplicate. All triplicates were consistent.
