# Supplementary material for: Partial Prion Cross-Seeding between Fungal and Mammalian Amyloid Signaling Motifs
Source: mBio. 2021 Feb 9;12(1):e02782-20. doi: 10.1128/mBio.02782-20 (PMC7885112; doi:10.1128/mBio.02782-20)
Supplement: Table S4 [file mBio.02782-20-st004.docx]

**Table S4A. Occurrence of the different phenotypes observed using fluorescence microscopy during co-expression experiments.**

| co-expressed transgenes | phenotypes | independent observations | number of cells observed |
| --- | --- | --- | --- |
| HET-s  HELLP(214-271) | [Het-s*] [π*] | 30 | 148 |
|  | [Het-s] [π*] | 14 | 124 |
|  | [Het-s*] [π] | 31 | 132 |
|  | [Het-s] [π] | 17 | 141 |
| HELLF(209-277) HELLP(214-271) | [φ*] [π*] | 18 | 85 |
|  | [φ] [π*] | 13 | 68 |
|  | [φ*] [π] | 17 | 87 |
|  | [φ] [π] | 18 | 82 |
| HELLP(214-271) CgHELLP(215-278) | [π*] [π*] | 6 | 36 |
|  | [π] [π] | 10 | 56 |
| PNT1(1-31) HELLP(214-271) | [π*] [π*] | 9 | 88 |
|  | [π] [π] | 12 | 71 |
| RIP3(444-469) RIP3(444-469) | [Rhim*] [Rhim*] | 2 | 14 |
|  | [Rhim] [Rhim] | 23 | 178 |
| RIP3(444-469) RIP1(524-551) | [Rhim*] [Rhim*] | 16 | 85 |
|  | [Rhim] [Rhim] | 27 | 123 |
| RIP3(4444-469) HELLP(214-271) | [Rhim*] [π*] | 5 | 27 |
|  | [Rhim] [π*] | 6 | 33 |
|  | [Rhim*] [π] | 3 | 18 |
|  | [Rhim] [π] | 32 | 170 |
| RIP1(524-551) HELLP(214-271) | [Rhim*] [π*] | 12 | 71 |
|  | [Rhim] [π*] | 6 | 36 |
|  | [Rhim*] [π] | 20 | 95 |
|  | [Rhim] [π] | 10 | 48 |

Prion phenotypes are defined by presence of foci of the corresponding fusion protein.

T**able S4B. Count of co-localized foci observed by fluorescence microscopy during co-expression experiments.**

|  |  |  |  | number of dots | | | |
| --- | --- | --- | --- | --- | --- | --- | --- |
|  |  |  |  | at least partially co-localized | | not co-localized | |
| co-expressed transgenes | co-expressed prions | independent observations | number of cells observed | n | % | n | % |
| HET-s  HELLP(214-271) | [Het-s] [π] | 17 | 141 | 50 | 5.1 | 925 | 94.9 |
| HELLF(209-277) HELLP(214-271) | [φ] [π] | 18 | 82 | 33 | 4.7 | 663 | 95.3 |
| HELLP(214-271)  CgHELLP(215-278) | [π] [π] | 10 | 56 | 428 | 90.5 | 45 | 9.5 |
| PNT1(1-31)  HELLP(214-271) | [π] [π] | 12 | 71 | 222 | 94.1 | 14 | 5.9 |
| RIP3(444-469)  RIP3(444-469) | [Rhim] [Rhim] | 23 | 178 | 524 | 96.0 | 22 | 4.0 |
| RIP3(444-469)  RIP1(524-551) | [Rhim] [Rhim] | 27 | 123 | 462 | 82.1 | 101 | 17.9 |
| RIP3(444-469)  HELLP(214-271) | [Rhim] [π] | 32 | 170 | 330 | 68.2* | 154 | 31.8 |
| RIP1(524-551)  HELLP(214-271) | [Rhim] [π] | 10 | 48 | 293 | 67.4* | 142 | 32.6 |

Counts of co-localized dots correspond to counts of foci with perfect or partial superposition of GFP and RFP whereas counts of not co-localized dots correspond to the number of isolated GFP dots plus the number of isolated RFP dots. * p-values were determined using two tails Fisher’s test by comparison of the number of co-localized dots in [Rhim] / [π] co-expressions to the number of not co-localized dots for non-cross interacting prion combination ([Het-s]/[π] or [φ]/[π]) and were <10^-10^).
